# Accessory regions and horizontal gene transfer shape the evolution of clonal *Colletotrichum nymphaeae* infecting strawberry

**DOI:** 10.1111/nph.71314

**Published:** 2026-06-05

**Authors:** Joris A. Alkemade, Alan G. Buddie, Anthony Kermode, Timothy G. Barraclough

**Affiliations:** ^1^ Department of Biology University of Oxford OX1 3EL Oxford UK; ^2^ Calleva Research Centre Magdalen College OX1 4AU Oxford UK; ^3^ Laboratory of Evolutionary Genetics, Institute of Biology University of Neuchâtel 2000 Neuchâtel Switzerland; ^4^ CAB International (CABI) SL5 7PY Ascot UK

**Keywords:** anthracnose, plant pathogen, population genomics, strawberry, transposons

## Abstract

- Rapid adaptation in fungal plant pathogens is often attributed to sexual recombination, yet many important pathogens are largely clonal. We investigated how genetic and phenotypic diversity arises in the predominantly asexual fungus *Colletotrichum nymphaeae*, the main cause of strawberry anthracnose in Europe and North America.
- We performed comparative genomics on 36 *C. nymphaeae* genomes and 45 other *Colletotrichum* genomes sampled from strawberry or from closely related species, assessing population structure, transposable element (TE) content, genome compartmentalisation and signatures of horizontal transfer, and linked these features to phenotypic variation and virulence.
- *Colletotrichum nymphaeae* consists of three major lineages, with a globally distributed clonal lineage showing high variability in morphology and virulence. Extensive variation in TE content was detected among and within lineages. Genomes are compartmentalised into core regions and TE‐rich accessory regions (ARs) that cluster by lineage and are enriched for gene duplications, genes under relaxed selection and genes linked to stress, virulence and fungicide resistance. We identified a *Starship* element and a 2 kb region containing two effector genes that were horizontally acquired.
- TE‐rich ARs and horizontal gene transfer drive diversification in this largely asexual pathogen, shaping its evolution and posing challenges for durable strawberry anthracnose management.

## Graphical abstract

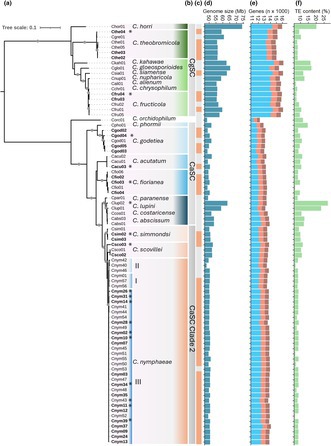

## Introduction

Fungal plant pathogens cause major crop losses world‐wide, threatening both food production and economic security (Savary *et al*., 2019; Fones *et al*., 2020). Disease control relies heavily on fungicides, host resistance and phytosanitary measures. However, the rapid adaptation of fungal populations and the emergence of new strains put continuing pressure on the effectiveness of current control strategies (Raza & Bebber, 2022). Especially in the past decades, crop resistance mediated by single *R* genes has been rapidly overcome, and newly introduced fungicides have lost efficacy at an accelerating pace (Dodds & Rathjen, 2010; Lucas *et al*., 2015; Fisher *et al*., 2018). Understanding how fungal pathogens adapt to and overcome control measures is therefore crucial for developing long‐term sustainable disease control strategies (Alkemade *et al*., 2025a).

To keep up with host resistance and external pressures, many fungi have evolved highly adaptable genomes. One key feature is the compartmentalisation of the genome into variable, transposable element (TE) and effector‐rich regions and more conserved, gene‐dense and TE‐poor regions, also referred to as the two‐speed genome (Dong *et al*., 2015; Torres *et al*., 2020). This compartmentalisation facilitates rapid diversification of effector repertoires, which often vary extensively among isolates and play key roles in host specificity and niche adaptation (Rovenich *et al*., 2014; van Dam *et al*., 2016; Badet *et al*., 2020; Le Naour‐Vernet *et al*., 2023). Genomic plasticity is further enhanced by accessory chromosomes (ACs) and large accessory regions (ARs) embedded within core chromosomes, which vary in presence or absence among isolates and can significantly contribute to adaptive potential and host range expansion (Ma *et al*., 2010; Möller & Stukenbrock, 2017; van Dam *et al*., 2017). In addition, fungal genomes often exhibit extensive variation in TE content, including large (> 5 kb) transposable elements known as *Starship* elements (Gluck‐Thaler *et al*., 2022; Bucknell & McDonald, 2023). Both TEs, especially *Tc1/Mariner* (TcMar) DNA transposons (Romeijn *et al*., 2025), and *Starships* have been implicated in horizontal gene transfer (HGT; Urquhart *et al*. 2024; Bucknell & McDonald, 2023), and could transfer virulence and antimicrobial resistance genes (Urquhart *et al*., 2022; Peck *et al*., 2024; Bucknell *et al*., 2025).

Many economically important fungal pathogens rely on sexual recombination to generate diversity. However, several major plant pathogens lack a known sexual stage and are predominantly represented by clonal lineages. Asexual reproduction enables rapid population expansion without the need for mating partners, contributing to their success as crop pathogens (McDonald & Linde, 2002). However, in theory, long‐term clonality limits adaptive potential and ultimately leads to extinction (Drenth *et al*., 2019; Bouvet *et al*., 2022). Despite this, asexual fungal pathogens often exhibit substantial inter‐ and intra‐lineage genetic diversity, suggesting alternative evolutionary mechanisms (Seidl & Thomma, 2014; Seidl *et al*., 2020; Alkemade *et al*., 2021, 2024). In the asexual pathogen *V. nonalfalfae*, adaptive evolution has been linked to the activity of a *Starship* element (Alkemade *et al*., 2025b), and *starship* elements appear to play a major role in *Verticillium* evolution in general (Sato *et al*., 2025). Likewise, the clonal pathogen *C. lupini* is highly enriched (*c*. 20%) in TEs, with considerable TE diversity observed within clonal lineages. In *F. oxysporum* and *Magnaporthe oryzae*, extensive variation and exchange of ACs are major drivers of adaptation among clonal strains (Barragan *et al*., 2024; van Westerhoven *et al*., 2025). Understanding how asexual fungal pathogens generate and maintain genetic diversity is therefore crucial for effective disease control.

Anthracnose disease is a major problem in strawberry (*Fragaria* × *ananassa* (Weston) Duchesne ex Rozier) cultivation and can cause severe yield loss (Howard *et al*., 1992). The disease is caused by members of two *Colletotrichum* species complexes, the *C. acutatum* Simmonds (*Ca*SC; Damm *et al*. (2012)) and *C. gloeosporioides* Penz species complex (*Cg*SC; Weir *et al*. (2012)). Within the *Cg*SC, *C. theobromicola* Delacr., also known as *C. fragariae* Brooks, was the first reported to cause anthracnose on strawberry in 1931 (Brooks, 1931), but *C. fructicola* Prihast and *C. siamense* Prihast are now the most reported pathogens on strawberry in China (Ji *et al*., 2022). Members of the *Ca*SC were first reported on strawberry in California (USA) in 1983 (Smith & Black, 1986), and have since spread world‐wide (Denoyes‐Rothan *et al*., 2003; Sreenivasaprasad & Talhinhas, 2005). From all *Ca*SC members, *C. nymphaeae* (Pass.) Aa (Damm *et al*., 2012) is the most widely reported pathogen on strawberry in North America and Europe (Baroncelli *et al*., 2015; Wang *et al*., 2019). Population studies based on few loci of *C. nymphaeae* suggest the pathogen to be clonal, with one lineage widespread across the globe (Baroncelli *et al*., 2015). As no highly resistant strawberry varieties are available due to breeding difficulties, disease control mostly relies on fungicides. The most commonly used fungicides in strawberry production are Azoxystrobin, a QoI fungicide targeting cytochrome *b* (*cytb*) gene (Forcelini *et al*., 2016), and azole fungicides targeting cytochrome P450 genes (Xu *et al*., 2014; Xiang & Zhang, 2018). However, over‐usage has led to widespread fungicide resistance (Forcelini & Peres, 2018; Wang *et al*., 2019). To improve disease management and resistance breeding, it is vital to understand the evolutionary dynamics of this destructive pathogen.

Despite the global importance of strawberry anthracnose, the biology and evolutionary dynamics of its causal agents remain poorly understood. In this study, we aim to (1) characterise the main causal agent of strawberry anthracnose disease and (2) investigate the contribution of transposable elements, accessory regions and HGT towards its adaptive potential related to virulence and antimicrobial resistance. To this end, we analyse genome assemblies from a historically and geographically diverse collection of *Colletotrichum* isolates obtained from strawberry. We hypothesise that *C. nymphaeae* is the predominant anthracnose‐causing species and seek to confirm its clonal population structure and that accessory genomic regions, transposable elements and HGT promote its diversification. Finally, we assess temporal genomic changes to identify signatures of adaptation that may inform future disease management strategies and strawberry breeding efforts.

## Materials and Methods

### Culture collection and sequencing

A total of 33 strawberry‐infecting *Colletotrichum* isolates, collected between 1967 and 2000 and representing 17 countries and 6 continents, were obtained from the CABI fungal culture collection (Supporting Information Table S1). The isolates were single‐spored and maintained on potato dextrose agar (PDA; Carl Roth, Germany) at 22°C in the dark as working cultures. Isolates were stored in 25% glycerol at −80°C for long‐term storage. To extract DNA, 10‐d‐old mycelium was frozen at −80°C and shredded for 40 s using a TissueLyser II (Qiagen, Hilden, Germany) at 30 Hz. Genomic DNA was isolated using a DNeasy® Plant Mini Kit (Qiagen) according to the manufacturer's instructions. Whole‐genome shotgun sequences were obtained through 150 bp paired‐end sequencing at a depth of > 80× coverage on an Illumina NovaSeqX Plus platform by Novogene (Cambridge, UK). Five isolates were additionally sequenced with Oxford Nanopore Technology (ONT; Oxford, UK). Libraries were prepared using the Rapid sequencing DNA V14—barcoding (SQK‐RBK114.24) kit according to the manufacturer's instructions. The libraries were loaded on R10.4.1 flow cells (FLO‐114) and sequenced for 48 h on a MinION Mk1D. Base calling and trimming were performed using Dorado v.0.5.3. For comparison, we included 19 publicly available *Colletotrichum nymphaeae*, 16 *Ca*SC and 13 *Cg*SC species genomes (Table S1).

### Genome assembly

Read quality was evaluated using FastQc v.0.11.9 (Andrews, 2010) and summarised using MultiQc v.1.14 (Ewels *et al*., 2016). Raw Illumina reads were trimmed for adapter sequences and filtered for a length of > 50 bp and an average Phred quality of > 20 using FastP v.0.23.4 (Chen *et al*., 2018). Raw ONT reads were filtered for a length of > 500 bp and an average Phred quality of > 10 using Chopper v.0.8.0 (De Coster & Rademakers, 2023). Illumina reads were assembled using SPAdes v.3.15.3 (‐‐careful; Prjibelski *et al*. (2020)), and resulting scaffolds were filtered for minimum length (500 bp) and min (10×) and max (1000×) coverage. ONT reads were assembled together with corresponding Illumina reads with SPAdes (‐‐careful, ‐‐nanopore; Antipov *et al*., 2016). Resulting assemblies were further scaffolded using Longstich (v.1.0.5; Coombe *et al*., 2021) and polished with ONT reads using Racon (v.1.5.0; Vaser *et al*., 2017) and with Illumina reads using two rounds of Pilon (v.1.23; Walker *et al*., 2014). Polished assemblies were further scaffolded with Ragtag v.2.1.0 (Alonge *et al*., 2022) based on reference CnymJS0361 and filtered for a minimum length of > 1000 bp. All assemblies were screened and cleaned from potential contamination using blastn (nt database) and Blobtools v.1.1.1 (Laetsch & Blaxter, 2017). Quast v.5.0.2 (Gurevich *et al*., 2013) was used to assess assembly quality, and genome completeness was estimated based on the presence of conserved single‐copy genes using Busco v.5.4.7 (Manni *et al*., 2021) with the *glomerellales_odb10* database. k‐mers were counted using Jellyfish v.2.3.0 (Marçais & Kingsford, 2011).

### Variant calling and population genetics

Variants were called by mapping Illumina reads to the reference genome Cnym01 using Bwa v.0.7.17 (Li, 2013). Resulting SAM files were converted to BAM files which were indexed and sorted using SAMtools v.1.16.1 (Danecek *et al*., 2021). Variant calling was performed using mpileup of BCFtools v.1.14 (Danecek *et al*., 2021), and variants were filtered for quality (Q20), minimum sequencing depth (2), mean sequencing depth (5), minor allele count (2), minor allele frequency (0.01), and missing data (0.95) using VCFtools v.0.1.16 (Auton & Marcketta, 2009).

Population structure of *C. nymphaeae* was analysed using principal component analysis (PCA), prcomp in R 4.5.1 (R Core Team, 2020) and discriminant analysis of principal components using dapc in adegenet (Jombart, 2008). The number of retained PCs was determined with xvalDAPC, and the optimal number of clusters was inferred using Bayesian and Akaike information criteria (BIC and AIC). Admixture and clustering were assessed with Admixture v.1.3.0 (Alexander *et al*., 2009) using 10 independent runs (K) with tenfold cross‐validation. Phylogeny was visualised using a neighbour‐network approach (neighborNet in phangorn (Schliep, 2011)). Multi‐locus genotypes (MLGs) were estimated with mlg.filter in poppr v.2.9.8 (Kamvar *et al*., 2014) using a Euclidean‐distance threshold defined by cutoff_predictor, and clone correction was applied using a threshold of 0.05. Diversity statistics and minimum spanning networks were generated with poppr.msn. Genetic variation within and among populations was quantified using AMOVA (Excoffier *et al*., 1992) with 1000 permutations using amova in poppr, pi‐nucleotide diversity was calculated with nuc_div in pegas v.1.3 (Paradis, 2010), and genetic differentiation was estimated using FST with genet.dist in Hierfstat (Goudet, 2005). Linkage decay was calculated with Plink v.1.9 (Chang *et al*., 2015), and the standardised index of association ($\bar{\mathrm{rd}}$) was computed using samp.ia in poppr.

### Genome annotation

Transposable elements were annotated and soft masked with the Earl Grey pipeline (Baril *et al*., 2024), using the clustered Mycomobilome v.1.1 database (Baril & Croll, 2026), removing putative spurious TE annotations (< 100 bp) and running Heliano v.1.3.1 for Helitron‐like element detection (Li *et al*., 2024). TE divergence landscapes were assessed by extracting mean Kimura 2‐Parameter (K2P) distances from Earl Grey output. TE insertions were called using ngs‐te‐mapper2 (Linheiro & Bergman, 2012) using Cnym01 as a reference. Sequencing reads were mapped against the consensus TE library, using a window of 100 bp, to identify reference and non‐reference TEs. *Starship* elements were identified for assemblies with an N50 higher than 0.75 mb using Starfish v.0.3.3 (Gluck‐Thaler & Vogan, 2024) with default parameters. Candidate *Starship*s were identified based on sequence similarity to curated reference elements and the presence of hallmark tyrosine recombinase (YR) ‘captain’ genes, using Blast v.2.12.0+ (Camacho *et al*., 2009). To reduce false positives, predicted elements were further validated through synteny analysis with closely related species using nucmer from MUMmer v.4.0.0 (Marçais *et al*., 2018), requiring alignments of ≥ 10 kb with > 80% nucleotide identity.

Gene annotation was performed with Funannotate v.1.8.17 (Palmer & Stajich, 2020), using transcripts of *C. nymphaeae* SA‐01 (Cnym50, Baroncelli *et al*., 2016) and Uniprot proteins of *C. abscissum*, *C. fioriniae*, *C. fructicola*, *C. kahawae*, *C. orchidophilum* and *C. scovillei* as evidence. Augustus v.3.5.0 (Keller *et al*., 2011) was pretrained on Cnym01. Predicted proteomes were assessed for completeness using Busco. The secretome was defined by proteins with a signal peptide but no transmembrane domain as predicted by Phobius v.1.01 (Käll *et al*., 2004), Tmhmm v.2.0 (Krogh *et al*., 2001), WoLF Psort (Horton *et al*., 2007) and SignalP v.6.0 (Teufel *et al*., 2022) integrated within Effhunter v.1.0 (Carreón‐Anguiano *et al*., 2020). Secreted proteins were screened for effector candidates by EffectorP 3.0 (Sperschneider & Dodds, 2022). Protein functions were predicted using Interproscan (Jones *et al*., 2014), Uniprot and Blast.

### Phylogenetic analysis

The phylogenetic relationship between the *Colletotrichum* species was determined using conserved single‐copy BUSCO genes and 1000 bootstraps with the BUSCO phylogenomics pipeline, available at https://github.com/jamiemcg/BUSCO_phylogenomics. Phylogenetic tree figures were rendered with iTol v.7 (Letunic & Bork, 2007).

*Starship* and TE phylogeny were based on pairwise distance matrix inferred by k‐mer comparisons with Mash v.2.3 (Ondov *et al*., 2016), with default k‐mer size of 21 and 10 000‐hash sketches. The resulting distance matrix was used to infer phylogenetic relationships using neighbour‐joining with 1000 bootstraps in R.

### Pangenome analysis

Core and accessory regions were identified in *C. nymphaeae* by performing all‐vs‐all whole‐genome alignments with Nucmer (max‐match), as described in van Westerhoven *et al*. (2024). We removed small alignments with the delta‐filter (‐l 5000) and only retained the best match per alignment (‐1). Genomic regions present in all 36 *C. nymphaeae* isolates were considered core, regions covered by < 80% were considered accessory, and regions between these values were considered softcore. Pairwise similarity between ARs was calculated using custom Python scripts (https://github.com/Anouk‐vw/Fusarium_pg), and alignments were visualised with Circlize (Gu *et al*., 2014). Orthologous groups (OGs) were identified with Orthofinder v.2.3.8 (Emms & Kelly, 2019). OGs present in all isolates were considered core, OGs found in > 28 genomes (*c*. 80%) were considered softcore, and genes found in < 28 of the genomes were considered accessory. Duplications with a support value higher than 50% were identified per OG. Nonsynonymous and synonymous substitutions (d*N*/d*S*) were determined per orthologous group by aligning all OG genes using Mafft v.7.520 (Katoh & Standley, 2013) followed by a codon‐guided nucleotide alignment, created by Pal2nal v.14.1 (Suyama *et al*., 2006), with CODEML (from Paml, v.4.9 (Yang, 2007)). Orthofinder was also used to identify OGs across all *Ca*SC and *Cg*SC isolates used in this study. A GWAS‐like approach was used to identify lineage‐specific OGs, using statgenGwas (van Rossum *et al*., 2020) and OrthoFinder‐generated gene‐count matrices.

### Temporal association

Temporal associations were performed using binomial regression models fitted independently for each SNP to evaluate changes in allele frequencies over time, following the approach described in Alkemade *et al*. (2025b). Analyses included only samples with non‐missing genotype calls at the focal site, with indels excluded. For each SNP *i*, the following model was fitted: $\log\left(\frac{p_{\mathrm{ij}}}{1-p_{\mathrm{ij}}}\right)=\beta_{i}^{0}+\beta_{i}^{1}x_{j}$, where $p_{\mathrm{ij}}$ denotes the probability that isolate *j* carries the alternate allele at SNP *i*, and $x_{j}$ represents the year of isolation of isolate *j*. The slope coefficient $\beta_{i}^{1}$ and its associated *P*‐value were extracted from each model to test for statistically significant temporal trends in alternate allele frequency (*P* < 0.01). Genes located within 5 kb upstream or downstream of significant SNPs, or residing on the same transposable element, were considered candidate genes.

### Expression analysis

RNA‐seq data from *C. nymphaeae* mycelium growing on PDA and from strawberry fruits and unwounded plants at 5 dpi from Ozbudak *et al*. (2025) were used to assess genome expression (Table S2). The genome of the isolate used in the experiments (02‐179) was not available, and Ozbudak *et al*. (2025) mapped the reads to isolate SA‐01 (Cnym50, III‐B), whose genome assembly is highly fragmented (1380 contigs; N50 = 91 kb). Raw reads were trimmed with Trimmomatic v.0.39 (Bolger *et al*., 2014), with a Phred score threshold of 33, a minimum read length of 50, and a sliding window of 5 : 10. Trimmed reads were mapped to the Cnym01 (I) and Cnym02 (III‐B) genomes using Star v.2.7.10b (Dobin *et al*., 2013), allowing for multiple read mapping and up to three mismatches (‐‐outFilterMultimapNmax 100, ‐‐winAnchorMultimapNmax 200, ‐‐outFilterMismatchNmax 3). Gene and TE counts were obtained using HTSeq‐count v.2.0.5 (Anders *et al*., 2015). Read counts were normalised and differentially expressed genes were identified with DESeq2 (Love *et al*., 2014). Given that only two biological replicates per treatment were available from Ozbudak *et al*. (2025), expression results should be interpreted with caution.

### Growth and virulence assays

Virulence and growth rate assays were conducted using isolates Clup02, Cfio03, Cfru04, Cgod04, Cnym02, ‐10, ‐11, ‐14, ‐26, ‐28, ‐31, ‐34, ‐43, ‐50, Csco03, Csim02 and Cthe04, representing *C. nymphaeae*, the *Ca*SC and the *Cg*SC (Table S1). Growth rate (mm d^−1^) was assessed by inoculating the centre of PDA plates with a 5 μl droplet of spore suspension (10^5^ spores ml^−1^) and incubating for 10 d at 22°C in the dark, with three replicates per isolate. Colony diameter was measured every 3 d, and plates were photographed from both sides after 10 d.

Virulence assays were performed on supermarket‐bought strawberries (cv Arwen, Fortuna and Gioelita) following Baroncelli *et al*. (2015). Fruits were surface‐disinfected for 5 min with NaDCC (0.5% active chlorine), rinsed with 70% ethanol and washed three times with sterile water. Up to six fruits were placed in sterile 1 l containers containing autoclaved sand to prevent fruit movement, wounded with a sterile needle and inoculated with 5 μl of spore suspension (10^6^ spores ml^−1^). Fruits were incubated for 5 d at 25°C and *c*. 70% relative humidity in the dark, after which lesion size was measured. All experiments followed a completely randomised design with at least five replicates.

### Statistical analysis

Statistical analyses were performed with R using the packages lme4 (Bates *et al*., 2015), lmertest (Kuznetsova *et al*., 2017) and emmeans (Lenth *et al*., 2017), stats and multcomp (Hothorn *et al*., 2012). Virulence data were analysed following a linear mixed‐effects model with isolate as fixed and variety, replicate and experiment as random factors. Differences between gene groups and lineages were analysed following a linear model. Characteristics that did not follow assumptions of normality of residuals and homogeneity of variance were square root or logit (percentage data) transformed. A Tukey‐HSD test (*P* ≤ 0.05) was applied for pairwise mean comparison of the different isolates and gene categories.

## Results

### Most isolates collected from strawberry are *Colletotrichum nymphaeae*

**Fig. 1:**
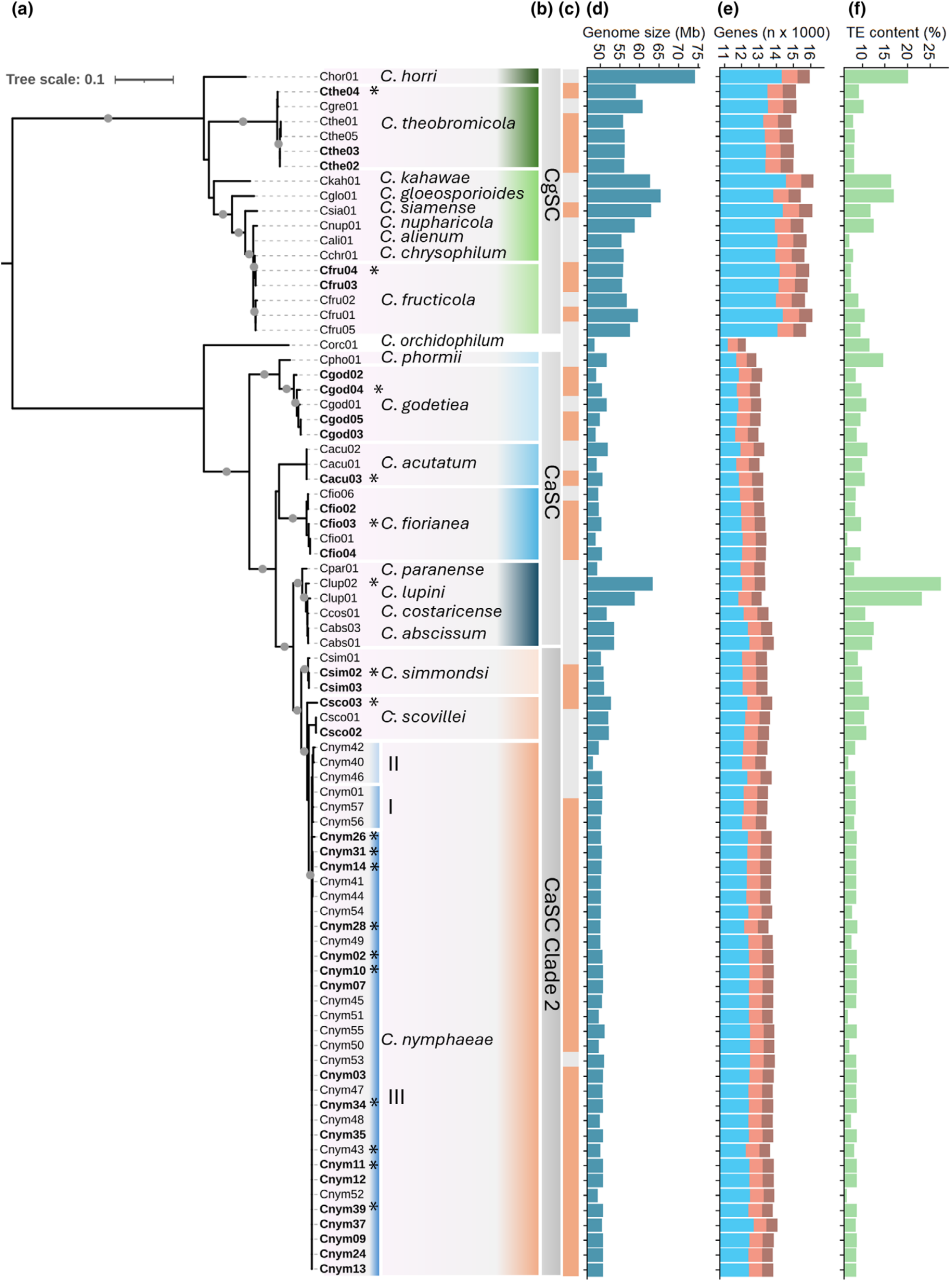
Genetic diversity of strawberry‐infecting *Colletotrichum* isolates. (a) Phylogenomic tree based on 6826 concatenated conserved single‐copy BUSCO genes estimated through maximum‐likelihood with 1000 bootstraps. Grey dots on nodes indicate bootstrap values above 80. Asterisks indicate isolates used for virulence assays. Latin numbers (I–III) indicate lineages within *Colletotrichum nymphaeae*. (b) Species complexes, *Cg*SC: *Colletotrichum gloeosporioides* species complex, *Ca*SC: *C. acutatum* species complex. (c) Collected from strawberry (red) or another host (grey). (d) Genome size in Mb. (e) Number of encoded genes (×1000) per isolate. Blue indicates non‐secreted, light red is secreted and dark red is predicted effector. (f) Transposable element content (%) per isolate.

A total of 33 *Colletotrichum* isolates collected from strawberry was obtained from the CABI culture collection and successfully sequenced and assembled using short‐read data (Table S1). Five *C. nymphaeae* isolates were additionally sequenced using ONT, resulting in long‐read assemblies (N50 > 3.8 Mb). Although short‐read assemblies were more fragmented (81–1624 contigs) compared to long‐reads (15–63 contigs), all assemblies contained at least 96.3% of the single‐copy BUSCO genes (Table S1). For comparative genomics, all publicly available *C. nymphaeae* genomes (19, accessed August 2025) and genomes of related species of the *Ca*SC (14) and *Cg*SC (13) were included, resulting in a dataset of 81 *Colletotrichum* genomes (Table S1). Phylogenomics based on 6825 BUSCO proteins showed that among the 33 sequenced CABI isolates, 17 belonged to *C. nymphaeae*, 1 to *C. scovillei*, 2 to *C. simmondsii*, 4 to *C. godetiae*, 3 to *C. fioriniae* and 1 to *C. acutatum* within the *Ca*SC (Fig. 1a; Table S1). Three isolates grouped with *C*. *theobromicola* and 2 with *C. fructicola* within the *Cg*SC. The *C. nymphaeae* genomes had a mean size of 50.3 Mb (48.2–51.1 Mb), with an average TE content of 8.3% (6.6–8.9%), and encoded an average of 13 763 genes (13 398 to 14 087), including an average of 628 (603–637) predicted effector candidates (Figs 1, S1). In comparison, other *Ca*SC assemblies exhibited a mean genome size of 51.4 Mb (48.6–63.4 Mb), with an average TE content of 9.9% (6.6–27.4%), and encoded a mean of 13 310 genes (12 242–13 860), of which 606 (458–647) were predicted effector candidates. The *CgSC* assemblies showed the largest mean genome size at 59.2 Mb (55.4–74.1 Mb), with an average TE content of 9.7% (7.1–20.1%), and encoded an average of 15 453 genes (13 554–16 164), including 738 (557–773) predicted effector candidates. As most of the strawberry infecting isolates belong to *C. nymphaeae*, we focus on this pathogen but included a wide sample of CgSC and CaSC taxa for context.

### Most *C. nymphaeae* isolates belong to a widely distributed clonal lineage

**Fig. 2:**
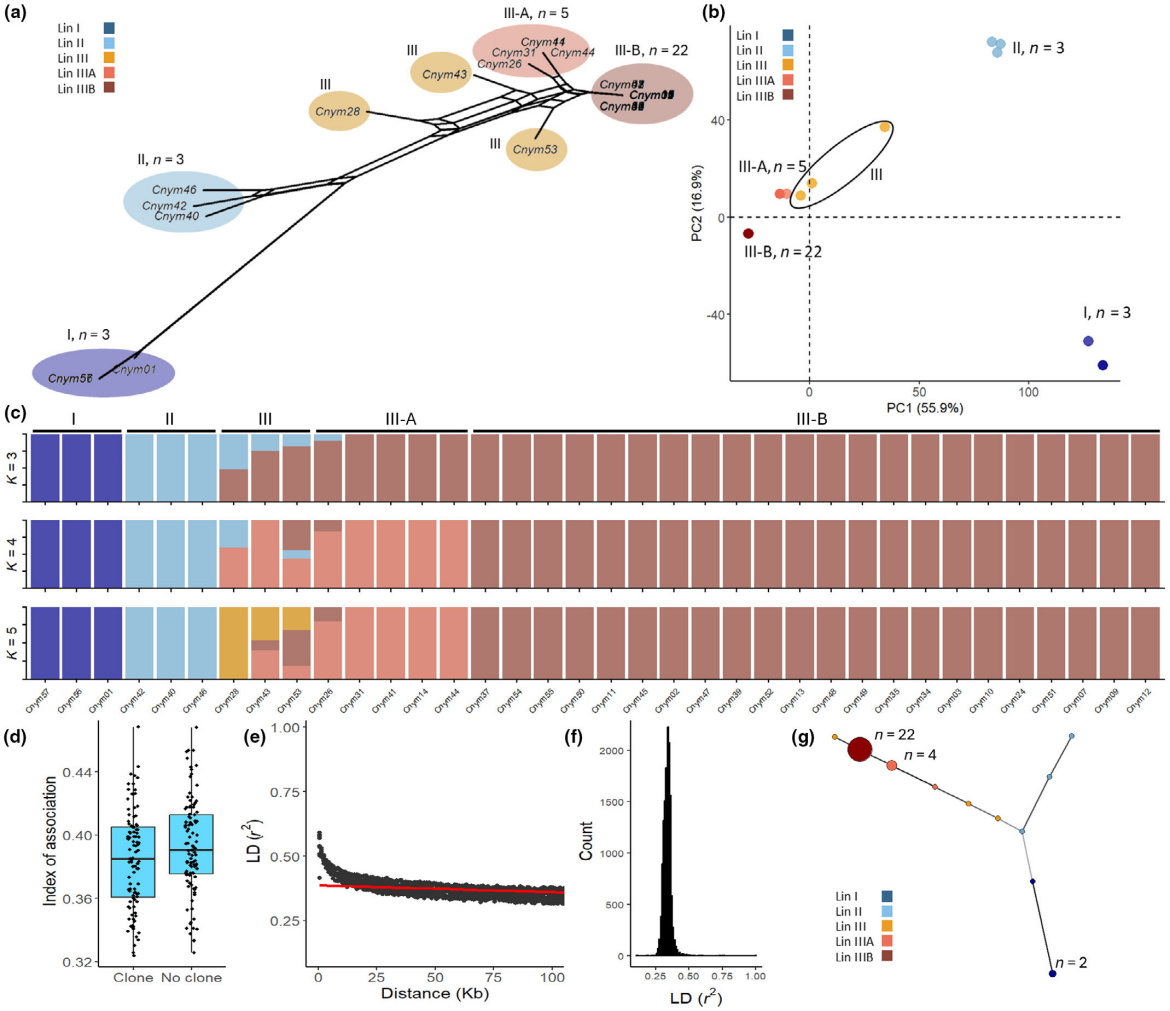
Population structure of *Colletotrichum nymphaeae*. (a) Network tree based on 42 715 SNPs called on reference genome Cnym01, showing three major groups (I–III) with two separate groups within lineage III (III‐A and III‐B). (b) Principal component analysis, dividing *C. nymphaeae* into five distinct lineages. (c) STRUCTURE analysis showing low admixture between *C. nymphaeae* lineages. (d) Index of association of clone and non‐clone‐corrected data, showing a mean of 0.41, indicating clonality. Horizontal line shows median, box shows interquartile range, whiskers extend to 1.5× interquartile range, all points are shown. (e) Linkage disequilibrium decay (LD) and (f) count of linked SNPs. (g) Minimum spanning network (MSN) showing multi‐locus genotypes of clone‐corrected data.

All *C. nymphaeae* isolates group into a single clade that can be divided into three low diversity lineages (I–III), with lineage II consisting solely of isolates not collected from strawberry (Fig. 1). Population structure analyses (network tree, Fig. 2a; PCA, Figs 2b, S2a,b; STRUCTURE, Fig. 2c; clustering, Fig. S3), based on 42 715 SNPs, confirmed these 3 major groups, but group III could be separated further into distinct groups with two clonal lineages (III‐A and III‐B) being the most common. Most isolates (*n* = 22, 61%) belong to lineage III‐B and were collected in Africa, Europe and the Americas between 1986 and 2015 (Fig. S4). Strong clustering, low admixture, low within‐group genetic diversity (Shannon‐Wiener Diversity Index (*H*) = 1.6, pi‐nucleotide diversity = 0.095; Table S3), a mean $\bar{\mathrm{rd}}$ of 0.41 and slow linkage disequilibrium (LD) decay (Fig. 2d–f), indicate a predominant asexual lifestyle. Lineage III‐A and III‐B appear to be fully clonal, showing low pi‐nucleotide diversity levels (III‐A: 0.03, III‐B: 3.63e‐4) and group III‐B showing only one, and group III‐A only two MLGs after clone correction (Fig. 2g).

The potential for sexual reproduction was additionally assessed by screening for genes associated with mating, including the MAT1 locus, which contains SLA2, the *Colletotrichum*‐specific MAT1‐2‐15, MAT1‐2‐1 and APN2, and mating pheromones with corresponding receptors (PPG1‐α:PRE2‐α and PPG2‐a:PRE1‐a; Wilson *et al*., 2021). The function of these pheromones is unclear in *Colletotrichum*, but in other ascomycetes, their expression is regulated by the MAT1 locus, and they play a role in mating type recognition. Loss of pheromone loci is therefore expected to correlate with impaired sexual function. The MAT1 locus was present in all analysed *Colletotrichum* species and was conserved across species complexes (Fig. S5). The a‐factor pheromone gene (PPG2‐a) was not detected in any genome, but its receptor (PRE1‐a) was present in all species outside the CaSC (Fig. S5a). The α‐factor pheromone and receptor (PPG1‐α:PRE2‐α) were identified in the CgSC and in CaSC clade 5, which includes one of the only CaSC species with an observed sexual morph *C. salicis* (Csal01; Damm *et al*., 2012). By contrast, the other CaSC species and *C. nymphaeae* only possessed the α‐factor pheromone with no receptor or lacked all pheromone system‐related genes, consistent with the lack of sexual reproduction in these species.

### Virulence and morphology vary among isolates and are not lineage‐specific

**Fig. 3:**
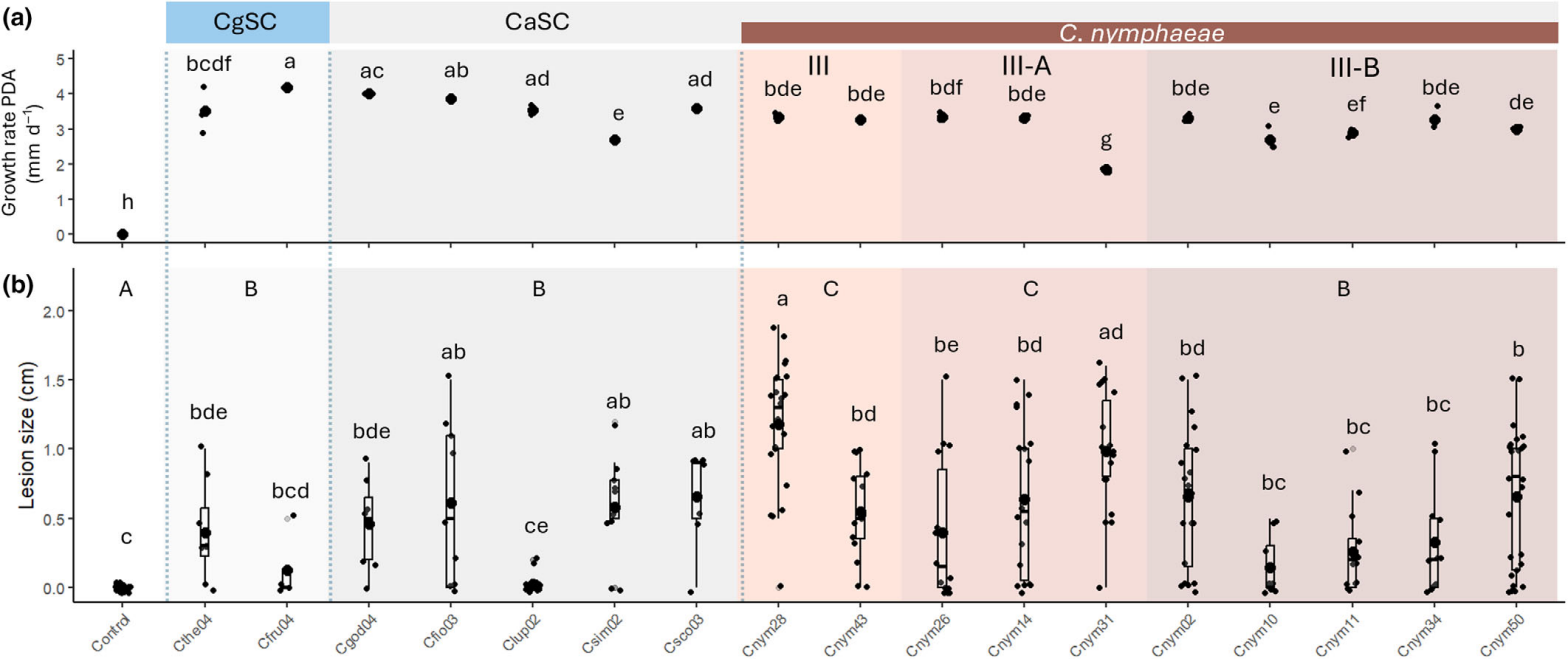
Growth rate and virulence on strawberry fruit of *Colletotrichum* species. (a) Growth rate in mm d^−1^. (b) Lesion size (cm) on strawberry fruit at 5 d postinoculation (dpi). Letters within plots indicate significant differences between gene categories (Tukey's test, *P* < 0.05). Large dot within boxplot indicates the mean, box shows interquartile range, whiskers extend to 1.5× interquartile range, all points are shown.

*Colletotrichum* species have been reported to show species or lineage‐specific virulence patterns, as seen for *C. lupini* (Alkemade *et al*., 2021) and *C. orbiculare* (Matsuo *et al*., 2022), so to assess isolate virulence, strawberry fruits were wound‐inoculated with a subset of isolates representing the different *C. nymphaeae* lineages and *Ca*SC, and *Cg*SC species (Fig. 3, Table S1). Lesion diameters were measured 5 d postinoculation (dpi). Most isolates were pathogenic on strawberry, except for *C. nymphaeae* lineage III‐B isolates Cnym10 (0.14 cm), Cnym11 (0.26 cm) and Cnym34 (0.33 cm), *C. fructicola* isolate Cfru04 (0.13 cm) and *C. lupini* isolate Clup02 (0.02 cm), which showed no increased lesion size relative to the control (Tukey‐test, *P* > 0.05). The *C. nymphaeae* isolate Cnym28 (lineage III) produced the largest average lesion size (1.18 cm), followed by Cnym31 (0.98 cm; III‐A). Among non‐*C. nymphaeae* isolates, *C. scovillei* (Csco03) caused the largest lesions (0.65 cm), comparable to *C. nymphaeae* isolates Cnym02 (0.65 cm; III‐B), Cnym50 (0.65 cm; III‐B) and Cnym14 (0.63 cm; III‐A). No significant lineage or species complex–specific effects were detected (ANOVA, *P* > 0.05), suggesting that virulence is not lineage nor species‐specific. Nevertheless, the overall mean lesion size was significantly, but only slightly, higher in lineage III (0.79 cm) and III‐A (0.81 cm) compared to III‐B (0.49 cm), the *Ca*SC (0.38 cm) and the CgSC average (0.31 cm; Tukey‐test, *P* < 0.05). Growth rates over 7 d showed the slowest growth (mm/day) for Cnym31 (1.8) and the highest for Cfru04 (4.2) and no correlation between virulence and growth rate was observed (Fig. S6; all isolates: *R* = −0.32, *P* = 0.23; within *C. nymphaeae*: *R* = −0.15, *P* = 0.68). Besides growth rate and virulence, isolate morphology within and between lineages was variable as well (Fig. S7). Despite the strong indication of clonality of lineage III‐A and B, virulence, isolate morphology and growth rates were not identical within clonal groups, suggesting other sources of diversity not apparent from SNP variation.

### Dynamic transposable element landscapes shape *Colletotrichum* genomes

**Fig. 4:**
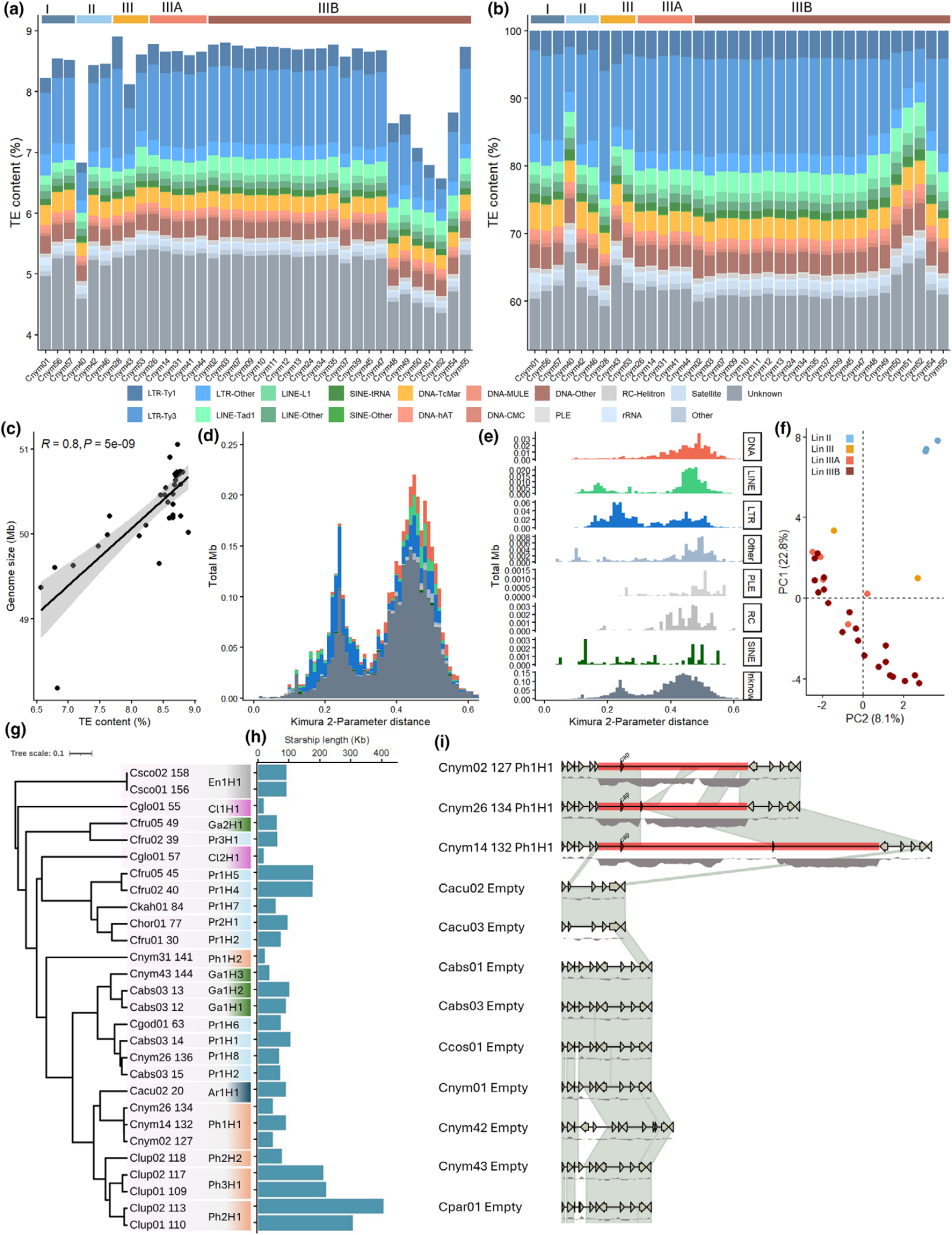
Transposable element (TE) landscape of *Colletotrichum nymphaeae*. (a) Contribution of TE subclass to genome size, legend is found below and bars on top indicate different lineages. (b) Proportion of TE subclass across all genomes, with 100% referring to the total TE content of the respective genomes. (c) Correlation (Pearson) of TE content (%) to genome size. Shaded region shows 95% confidence interval on the slope. (d) TE divergence (kimura 2‐parameter distance) landscapes across *C. nymphaeae* genomes, with the *y*‐axis showing genomic proportion (Mb) occupied by each TE class and the x‐axis indicating K2P distance. Peaks represent bursts of TE expansion, where younger elements (< 0.1) indicate recent activity while older elements (> 0.1) reflect ancestral bursts. TE class colours are presented in (e), showing TE divergence landscapes for each TE class separately. (f) Principal component analysis (PCA) based on the presence/absence matrix of transposable element (TE) insertions in reference genome Cnym01. (g) Phylogeny of *Starship* elements that occur in *Colletotrichum* species of the *acutatum* and *gloeosporioides* species complex, based on *k*‐mer similarity. Scale bar indicates the Mash distance that represents the *k*‐mer difference. *Starship* families and haplotypes are highlighted with colour bars. (h) Length of respective Starship elements in kb. (i) Similarity of Ph1h1 *Starships* and surrounding regions between closely related *Colletotrichum* species. Blue density plot indicates TE content.

Transposable element (TE) content across all analysed *Colletotrichum* species showed a strong positive correlation with genome size (*R* = 0.64, *P* = 3.8 × 10^−11^; Fig. S8c). This relationship was even stronger within the *C. acutatum* species complex (CaSC; *R* = 0.93, *P* < 2.2 × 10^−16^; Fig. S8d) and was also observed within *C. nymphaeae* (*R* = 0.8, *P* = 5 × 10^−9^; Fig. 4c). The highest TE content was observed in *C. lupini*, ranging from 23% to 27%. This high content is largely attributed to relatively recent bursts of long terminal repeat (LTR) retrotransposons (K2P distance = 0.17), which account for 55% of the total TE content in this species (Fig. S8b).

In *C. nymphaeae*, TE content ranged from 6.6% to 8.9% (Fig. 4a), with no lineage‐specific patterns detected (Fig. S1). Moreover, TE content did not correlate with isolate virulence (*R* = −0.026, *P* = 0.94; Fig. S9), nor with assembly quality metrics, including genome coverage (*R* = 0.055, *P* = 0.75), number of contigs (*R* = −0.14, *P* = 0.4) or number of uncalled bases per 100 kbp (Ns/100 kbp; *R* = 0.023, *P* = 0.9), suggesting minor influence of sequencing strategy on TE annotation. The greatest variability in TE content was observed within clonal lineage III‐B, where values ranged from 6.6% to 8.8%, highlighting substantial variation among clonal isolates (Figs 4a, S1). This variability is further supported by the presence/absence polymorphisms of TE insertions relative to the reference genome Cnym01. These patterns reveal lineage III(A & B) as a genetically diverse group rather than three distinct clusters, suggesting that many TE insertions are recent, dynamic and not fixed across lineages (Fig. 4f).

Within *C. nymphaeae*, unclassified (‘unknown’) elements constituted the largest TE category, accounting on average for 61.7% of total TE content (range: 59.2–67.2%), underscoring the limited characterisation of fungal TEs (Fig. 4b). Among classified elements, LTR/Ty3 (formerly known as Gypsy (Wei *et al*., 2022)) elements were the most abundant (mean: 12.6%, range: 5–16%), followed by LTR/Ty1 (also known as Copia) elements (3.9%) and DNA/TcMar transposons (3.2%). The longest elements were LTR/Ty3 (1022 bp), LTR/Ty1 (826 bp) and LINE/Tad1 (761 bp; Fig. S10). Lineages I and II exhibited shorter LTR/Ty1 elements on average (640 bp) compared to lineage III (A and B; 886 bp; Fig. S11), while lineage I harboured the longest DNA/MULE‐MuDR elements (692 bp vs 479 bp in other groups).

Analysis of TE divergence landscapes in *C. nymphaeae* revealed two major historical bursts (Fig. 4d). An ancient burst (K2P distance 0.32–0.6) was dominated by unknown elements (67%), whereas a more recent burst (K2P distance 0.1–0.32) still contained a high proportion of unknown elements (50%) but also included a substantial fraction of LTR retrotransposon elements (39%). No pronounced peak of recent activity (K2P < 0.1) was detected; however, this part showed a tenfold enrichment in SINE elements compared to older peaks (Fig. 4e). A similar temporal pattern was observed across all analysed *Colletotrichum* species. A major ancient burst (K2P > 0.32) was primarily composed of unknown elements (65%), while more recent bursts (K2P 0.1–0.32) were enriched in LTR elements (49%; Fig. S8e). The most recent insertions (K2P < 0.1) were dominated by DNA transposons (27%; Fig. S8f). When individual *C. nymphaeae* isolates were analysed separately, a pattern of rapid LTR accumulation over a relatively short evolutionary timescale (K2P < 0.3) became apparent, suggesting that LTR elements have played a key role in the species' recent evolutionary history.

### Lineage III‐A and B isolates share same TE‐rich *Starship* element

Giant transposable elements referred to as *Starships* were identified from all high‐quality *Colletotrichum* assemblies available in this study (N50 > 0.75 Mb, Table S1) using Starfish (Gluck‐Thaler & Vogan, 2024). Starships were confirmed through visual inspection of alignments with closely related species. We identified 28 *Starships* that belong to 24 haplotypes of 12 naves of 6 families, ranging between 19 and 407 Kb (Fig. 4g,h; Table S4). Within *C. nymphaeae Starships* were identified in Cnym02 (III‐B), Cnym14 (III‐A), Cnym26 (III), Cnym31 (III‐A) and Cnym43 (III‐B). No *Starships* were identified in Cnym01 (I), 28 (III) and 42 (II). The same *Phoenix Starship* (Ph1Hp1) was found on chromosome 1 of Cnym02, Cnym14 and Cnym26, ranging between 48.5 and 91.5 kb (Fig. 4i), but was not found in the other lineage III‐A isolate Cnym31. Apart from the captain YR, no protein‐coding genes were identified on the Cnym02 element, but one gene encoding for an AC transposase was identified on the Cnym14 and Cnym26 element. Despite being gene poor, these *Starships* were highly enriched with TEs (> 80%), with the majority being LTR/Ty3 (33%) and LTR/Ty1 (35%) elements. Similar *Phoenix Starship* elements were identified in Cnym31 (IIIA; Ph1Hp2) and in the asexual lupin pathogen *C. lupini* (Ph2Hp1, Ph2Hp2 and Ph3Hp1; Fig. 4g). The isolate Cnym26 showed an additional *Starship* of the *Prometheus* family on chromosome 9 with a length of 67.7 kb, similar to elements found in Cabs03 and Cgod01. This element contained 12 genes (Table S5), encoding for a C2H2 finger domain‐containing transcription factor (Cnym26_010720) and a serine/threonine protein kinase (Cnym26_010727), which are associated with virulence in *Colletotrichum* (de Oliveira Silva *et al*., 2024; Fu *et al*., 2024).

### Temporal change within clonal group III‐B associates with *Starship* element

To identify potential signatures of temporal adaptation, we tested for associations between genetic variation and year of isolation across *C. nymphaeae* isolates collected over a 50‐yr period (1977–2017). Temporal association analyses were performed using binomial regression, with SNP presence/absence as the response variable and sampling year as the explanatory variable, following the approach described in Alkemade *et al*. (2025b). Using Cnym01 (lineage I) as the reference genome, this analysis identified a single SNP significantly associated with time (Cnym01_1_2204686; *P* = 0.02; Table S6), located within a gene encoding tRNA 4‐demethylwyosine synthase (Cnym01_000556; Table S5). When using Cnym14 (III‐A) as the reference genome, one significant association was detected on chromosome 8 (Cnym14_8_1431272; *P* = 0.016), located near a chromosome segregation protein gene (*Cse1*). Using Cnym02 (III‐B) as the reference genome yielded two significant associations: one SNP on chromosome 6 (Cnym02_6_262916; *P* = 0.015) located 2450 bp from a gene encoding an immunogenic protein, and a second SNP on chromosome 8 (Cnym02_8_1443354; *P* = 0.016) located near a *Cse1* homolog, corresponding to the association identified using Cnym14. The strongest temporal associations (*P* < 0.01) were observed within clonal lineage III‐B. In this group, 13 SNPs on chromosome 1 of Cnym02, spanning positions 5429 163 to 5429 494 (Table S6; Fig. S13), showed significant associations with time. These SNPs are located within the previously identified *Phoenix Starship* element s127 (Ph1h1; Fig. 4f–h) and within an unclassified TE element (MYCMOB1.1_FAMILY‐174 522‐COLLUP_NE_1662). This element appears to be unique to lineage III‐B and A isolates in *C. nymphaeae* but is shared with *C. lupini* and *C. costaricense* (Fig. S14), with the *C. lupini* elements also located within *Phoenix Starships*. Together, these observations suggest horizontal transfer and a potential role in the recent temporal adaptation of this *Starship* and unclassified TE element.

### Accessory regions cluster with TEs indicating genome compartmentalisation

**Fig. 5:**
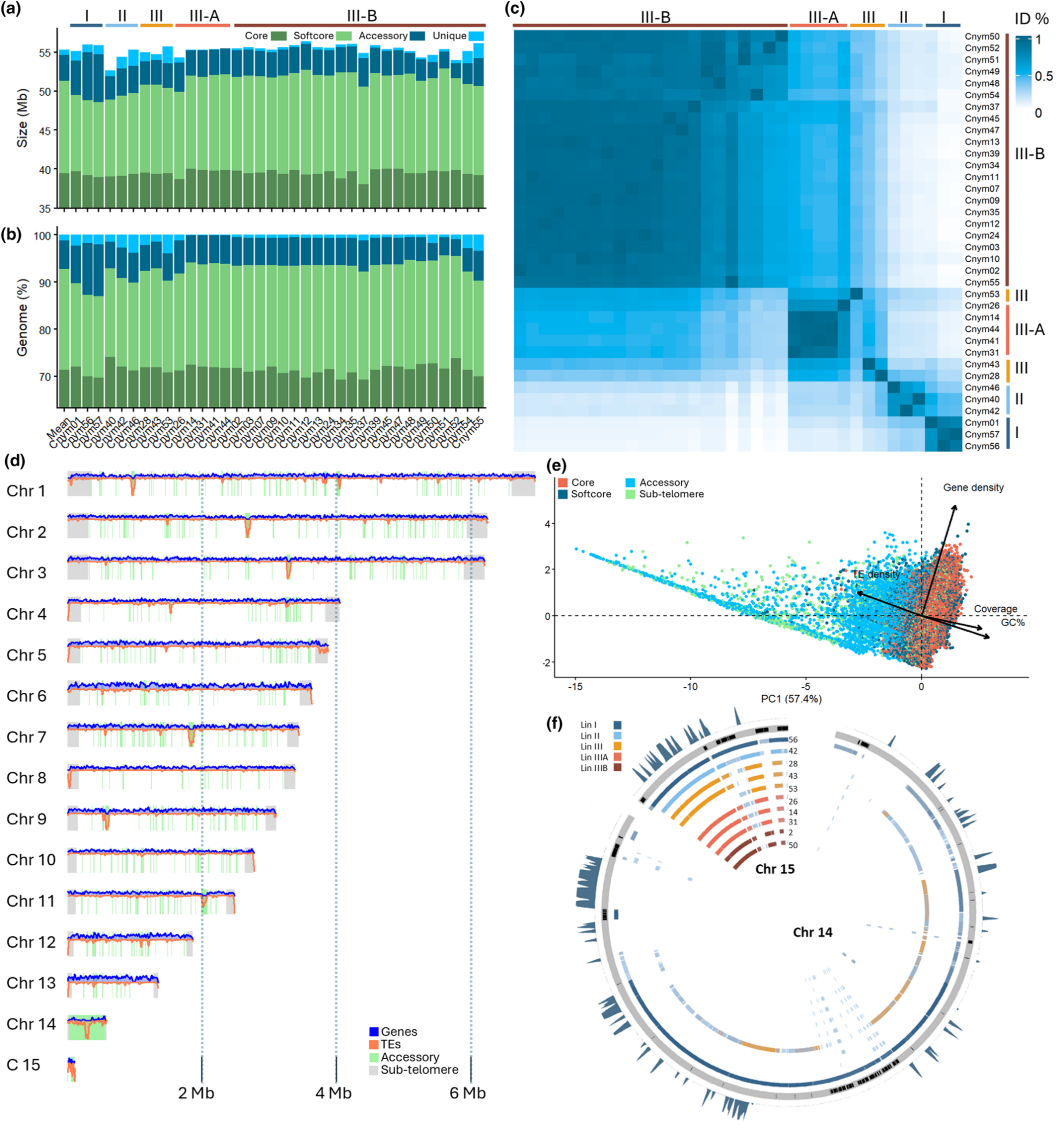
Accessory regions (ARs) within *Colletotrichum nymphaeae* and Cnym01. (a) Genome sizes in Mb of *C. nymphaeae* genome divided by core (present in all), softcore (present in > 80%), accessory (present in < 80%) and unique regions. (b) Proportion of each genome allocated to core, softcore, accessory and unique regions. (c) Assigned *C. nymphaeae* lineages group together based on AR identity. ARs seem highly lineage‐specific, sharing little with other lineages. (d) Genome overview of Cnym01, showing ARs (light green) are clustered together with transposable elements (TEs; bottom red line) rich and gene (upper blue line) poor regions. A similar pattern is found for the sub‐telomeric regions (grey, first and last 5% of the chromosome). (e) This pattern is further accentuated by principal component analysis (PCA) on gene‐, TE‐, GC content and coverage of 36 *C. nymphaeae* isolates, clustering ARs (light blue) and sub‐telomeric regions with high TE density and core (dark red) and softcore (red) regions with high gene density and coverage. (f) Mini‐chromosomes 14 and 15 of Cnym01 (grey line). ACs 14 is only shared within lineage I, and partly with Cnym43 (III). Both chromosomes are gene‐poor (blue density plot) and TE‐rich (black).

ARs are often considered cradles of genetic diversity in fungal genomes. To identify ARs in *C. nymphaeae*, we compared *de novo* long‐read assemblies of multiple isolates to the reference genome Cnym01 (I) and performed an all‐vs‐all whole‐genome alignment across all assemblies. Overall, *C. nymphaeae* genomes exhibited high levels of synteny. Thirteen homologous chromosomes were conserved between Cnym01 and Cnym28 (III) and 26 (III; Fig. S15a,b). However, several structural differences were observed. In Cnym14 (III‐A) and Cnym31 (III‐A), chromosome 2 corresponded to chromosomes 4 and 11 of Cnym01, while chromosome 5 of Cnym14 and Cnym02 (III‐B) aligned to chromosomes 6 and 12 of Cnym01, suggesting either chromosomal rearrangements or misassembly (Fig. S15c–e). The number of accessory contigs (> 50 kb) and ACs (> 250 kb) ranged between one and four, with two (chromosome 14 and contig 15) for Cnym01, one (contig 14) for Cnym28, four (contig 14–17) for Cnym26, three (contig 12–14) for Cnym14, two for Cnym31 (contig 13–14) and four (contig 13–16) for Cnym02 (Fig. S15f). To further characterise genomic variation, we classified genomic regions into core (present in all isolates), softcore (present in ≥ 80%), accessory (present in < 80%) and unique (isolate‐specific) categories. On average, 71.4% of the *C. nymphaeae* genome was core, 21.4% softcore, 6.0% accessory and 1.2% unique (Fig. 5b). Accessory regions showed strong lineage specificity, with isolates clustering according to their lineage (Fig. 5c). Notably, ARs of lineage III (including III‐A and III‐B) did not overlap with those of lineages I and II. Across the genome, ARs were characterised by high transposable element density and low GC content and occurred in discrete genomic patches, indicative of a compartmentalised genome architecture (Figs 5d,e, S16).

### Accessory regions contain fungicide targets and stress‐related genes

The largest AR in Cnym01 is AC 14 (0.5 Mb), which is only shared with other lineage I isolates (Cnym56 and 57) and partly with Cnym43 (III), has a TE content of 25% and GC content of 46.8% (Fig. 5f). The chromosome contains 56 protein‐coding genes, with 77% being accessory, of which 25% are duplications. One of those duplications is a gene encoding a Cytochrome P450 (Cnym01_013739, Table S5), which is an important azole fungicide target (Yoshida, 1988). The mini‐chromosome consists mostly of genes encoding uncharacterised proteins, only two predicted effector candidates, and a few genes related to detoxification and stress regulation, such as major facilitator superfamily (MFS), pectin‐lyase and C2H2‐type zinc finger encoding genes (Table S5). Smaller ARs were found throughout the genome, with the largest found on chromosome 11 (0.08 Mb), 7 (0.08 Mb) and 2 (0.07 Mb), all having a high TE (> 71%) and low GC content (< 31%; Fig. 5d). In Cnym02, the largest AR (0.09 Mb) was found on chromosome 7 and had high TE (35%) and low GC content (29%; Fig. S16a). Accessory contigs 14 (0.07 Mb) and 15 (0.07 Mb) showed slightly lower TE content (< 12%) and an average GC content of 52%. Similar to AC 14 of Cnym01, Cnym02 contig 14 contained a lineage III‐B specific Cytochrome P450 (Cnym02_013974) and C2H2‐type zinc finger encoding genes (Table S5). In Cnym14, contig 12 is the largest AR (0.2 Mb), and showed a low TE content of 6% and GC content of 52%, which is in contrast with the other 3 large ARs on chromosome 7 (0.12 Mb, TE = 50%, GC = 31%), contig 14 (0.1 Mb, TE = 34%, GC = 44%) and the *Phoenix Starship* element on chromosome 1 (0.09 Mb, TE = 58%, GC = 31%; Fig. S15b). Accessory contig 16 (60 kb) of Cnym26 appears to be unique to this lineage and is enriched in genes encoding hypothetical proteins, one effector and a few genes related to detoxification, such as MFS encoding genes (Fig. S16j; Table S5). Together, these findings demonstrate that accessory regions in *C. nymphaeae* vary markedly in size, composition and gene content across lineages, and are enriched for genes associated with detoxification, virulence and fungicide resistance, underscoring their potential role in lineage‐specific adaptation.

### Accessory regions highly contribute to genome diversification

**Fig. 6:**
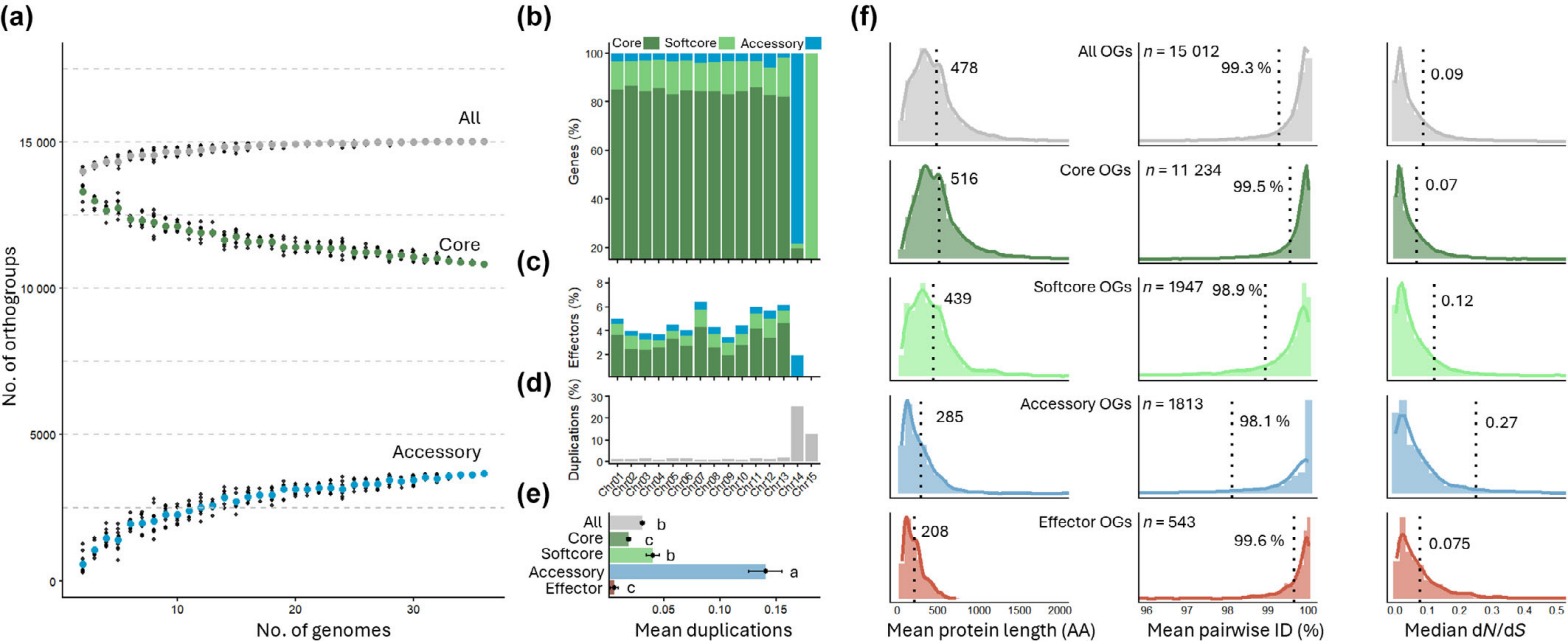
Pangenome and evolutionary dynamics of *Colletotrichum nymphaeae*. (a) Saturation of pangenome. Black dots show separate genome values, coloured dots show mean across samples: Grey = all, green = core, blue = accessory. (b) Distribution of core, softcore, accessory genes and (c) effectors per chromosome of Cnym01. (d) Proportion of duplicated genes per Cnym01 chromosome. (e) Mean duplication events per region type. Letters denote groupings of significantly different sets based on Tukey test. (f) Mean protein length (AA), mean pairwise identity (%) and median d*N*/d*S* of all, core, softcore, accessory and effector orthogroups (OGs). Dotted lines indicate the mean.

To characterise gene variation within *C. nymphaeae*, we performed a pangenome analysis on the available 36 genomes, grouping 494 337 predicted protein‐coding genes into 15 015 orthogroups (OGs). The pangenome comprised 11 234 core OGs (74.8%) present in all isolates, 1947 softcore OGs (13.0%) present in at least 80% of genomes and 1813 accessory OGs (12.1%) present in fewer than 80% of genomes. No isolate‐specific (unique) OGs were detected (Fig. 6a,b). Clustering based on accessory OGs recapitulated the previously defined *C. nymphaeae* lineages (Fig. S17). Pangenome size achieved saturation after > 30 genomes, indicating our collection captures most of the diversity of protein‐coding genes within the species (Fig. 6a). Among all OGs, 543 consisted exclusively of predicted effector genes, of which 151 (28%) were classified as accessory. In the reference genome Cnym01, conserved chromosomes (1–13) contained on average 84% core genes, 12% softcore genes and 3% accessory genes, although chromosome 12 showed a slightly elevated accessory gene content (6%; Fig. 6b). By contrast, ACs 14 was highly enriched for accessory genes (78%), while contig 15 consisted entirely of softcore genes. The average effector gene content of core chromosomes was 4.7%, with chromosomes 7, 11, 12 and 13 showing higher proportions (5.6–6.4%; Fig. 6c). ACs 14 contained fewer effectors overall (2.6%), all of which were accessory. Gene duplication rates varied across genomic compartments, with low rates on core chromosomes (1.1%) and markedly higher rates on ACs 14 (25%) and 15 (12.5%; Fig. 6d). Across the pangenome, accessory OGs (0.14) exhibited significantly higher duplication rates than core OGs (0.02; Tukey test, *P* < 0.05; Fig. 6e). Accessory genes were significantly shorter compared to core genes (mean length 285 vs 510 AA; Tukey test, *P* < 0.001), showed lower pairwise amino acid identity (98.1% vs 99.6%; *P* < 0.001), and evolved under more relaxed selective pressure (mean d*N*/d*S* = 0.27 vs 0.06; *P* < 0.001; Fig. 6f). Predicted effector OGs were even shorter on average compared to accessory OGs (208 AA; *P* < 0.001), but were more conserved (99.6% identity) and evolved under selective pressures similar to those of core OGs (d*N*/d*S* = 0.075).

### Accessory genes and TEs are transcriptionally activated during infection

Publicly available RNA‐seq reads from strawberry fruit and plant infection of Ozbudak *et al*. (2025) were mapped to the genomes of Cnym01 (lineage I) and Cnym02 (lineage III‐B). This resulted in incomplete gene coverage, with 11.3% of Cnym01 genes (including 50% of accessory genes) and 6.4% of Cnym02 genes (including 12.5% of accessory genes) unmapped. For both genomes, only 30% of annotated TEs were mapped, and the *Starship* element in Cnym02 was not detected. Despite these limitations, TEs showed de‐repression during strawberry plant infection, with stronger induction in leaf‐infection than in fruit infection (Fig. S18). Accessory genes and predicted effectors were strongly upregulated during plant infection (Fig. S19). Notably, accessory contig 14 of Cnym02 exhibited high expression during both plant and fruit infection (Fig. S20). Together, these results suggest that, despite incomplete mapping, TEs, effectors and accessory genes are transcriptionally activated during infection and likely play important roles in host colonisation.

### Two lineage III‐B specific effectors were horizontally acquired from the *Cg*SC

**Fig. 7:**
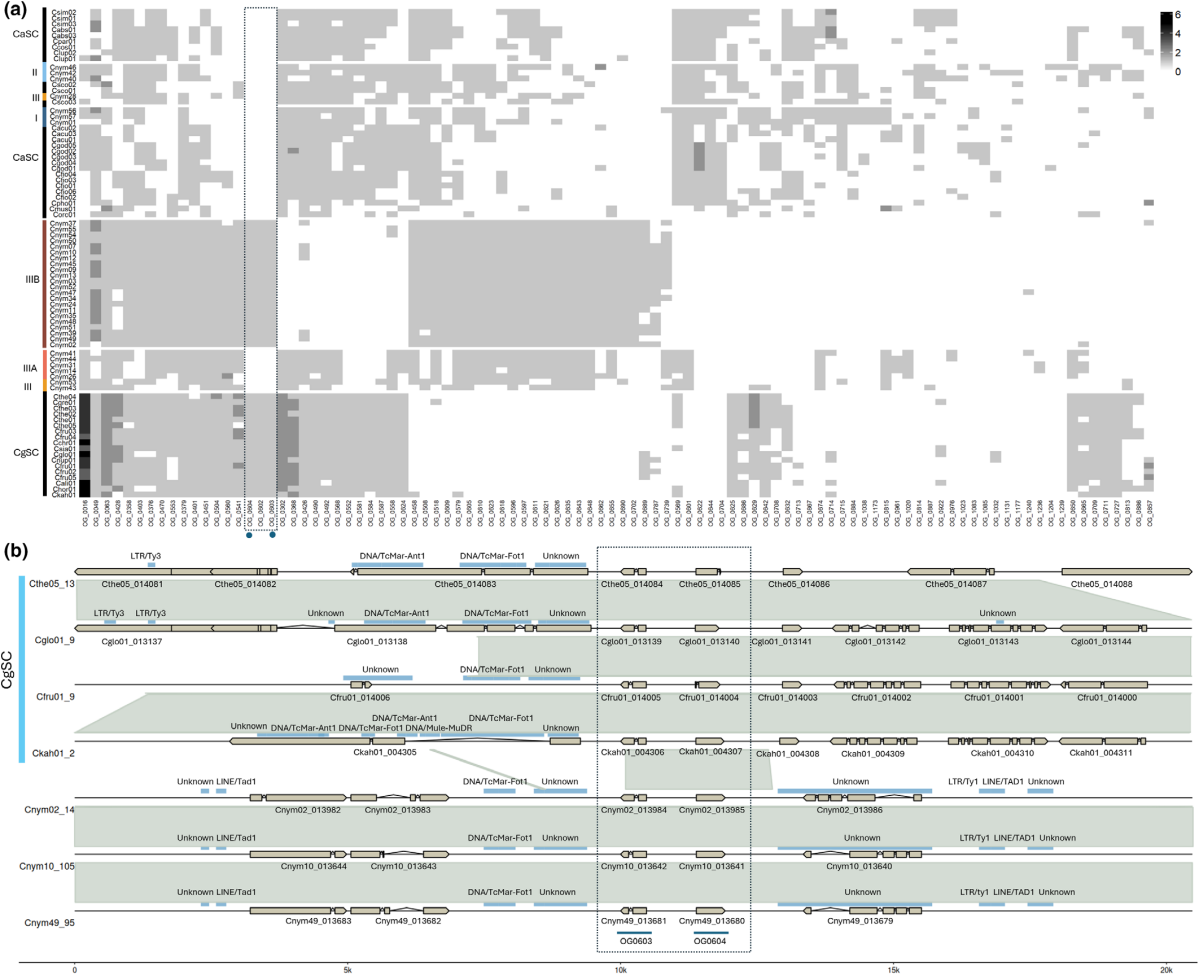
Lineage‐specific effectors and horizontal gene transfer between lineage III‐B and the *Colletotrichum gloeosporioides* species complex. (a) Effectors shared among *Colletotrichum nymphaeae*, the *Colletotrichum acutatum* species complex (CaSC), and the *C. gloeosporioides* species complex (CgSC). Effectors OG0603 and OG0604 (highlighted with blue dots) are unique to lineage III‐B and are also present in CgSC species. (b) Evidence of horizontal gene transfer (HGT) between the CgSC and *C. nymphaeae* lineage III‐B. A 20 kb genomic region was aligned across *C. theobromicola*, *C. siamense*, *C. fructicola* and *C. nymphaeae* (lineage III‐B), with isolate and chromosome/contig identifiers indicated. Overall synteny between *C. nymphaeae* and CgSC species is low; however, a conserved *c*. 2 kb region (within the dashed box) contains the two aforementioned effector genes, encoding a secreted protein and a hypersensitive response‐inducing protein (Supporting Information Table S4). These genes are conserved within *C. nymphaeae* lineage III‐B and across CgSC species (Fig. S22). A DNA/TcMar transposable element is located 1.3 kb downstream of Cnym02_013984 and may have facilitated the HGT event. HGT figure was rendered with gggenomes v.1.1.3 (Hackl *et al*., 2024).

Effectors play an important role in defining host range and we expected strawberry infecting *Colletotrichum* isolates to cluster together based on presence‐absence profiles. For the 81 *Colletotrichum* isolates used in this study we predicted a total of 52 258 effector candidates which could be grouped into 1245 OGs. Clustering showed that isolates clustered with species and lineage rather than host (Fig. S21). Zooming in on lineage‐specific effector candidates, using a GWAS‐like approach (Alkemade *et al*., 2024), further accentuated lineage‐specific profiling (Fig. 7a). Three lineage III‐B specific OGs were conserved among species of the distantly related *Cg*SC. The three OGs contain genes encoding an uncharacterised protein (OG0602), a secreted protein (OG0603) and a hypersensitive response‐inducing protein (OG0604, Fig. 7a, Table S5). OG0603 and OG0604 correspond to the Cnym02 genes Cnym02_013984 and Cnym02_013985, respectively, and are both located on accessory contig 14. This contig is highly upregulated during strawberry plant and fruit infection (Fig. S20). Notably, these genes share homologs with members of the *Cg*SC (Fig. S22) and reside within a 2 kb genomic region which is conserved across the *Cg*SC, providing strong evidence for HGT (Fig. 7b). Another lineage‐specific effector that might have transferred horizontally was identified for lineage I (OG_1173; Fig. 7a), encoding a non‐reducing end alpha‐l‐arabinofuranosidase likely involved in hemicellulose binding (Miyanaga *et al*., 2006), with homologs in other strawberry anthracnose‐causing species such as *C. scovillei*, *C. simmondsii*, *C. fructicola* and *C. siamense*. We also identified OGs with effector genes unique to *C. nymphaeae*, with OG_0655, representing a lineage III‐A and B effector candidate encoding an AA1‐like domain‐containing protein, and OG_0648 specific to lineage III, encoding an uncharacterised effector (Table S5). These results suggest that HGT and accessory genomic regions appear to play key roles in the emergence and maintenance of lineage‐specific effector repertoires.

## Discussion

Anthracnose is a destructive strawberry disease caused by several *Colletotrichum* species (Ji *et al*., 2022). Whole‐genome sequencing of 33 strawberry‐infecting isolates confirmed *C. nymphaeae* as the primary cause of strawberry anthracnose in Europe and North America (Baroncelli *et al*., 2015; Wang *et al*., 2019). Historically, the disease was attributed to *C. acutatum* (Sreenivasaprasad & Talhinhas, 2005), but many of these records likely represent misidentified *C. nymphaeae* (Damm *et al*., 2012; Baroncelli *et al*., 2015), with implications for phytosanitary regulation, as only *C. acutatum* was listed as a quarantine organism in the UK and EU (Calleja *et al*., 2013). No strawberry‐infecting isolates from Asia were available for this study, but the major species seem to be *C. siamense* and *C. fructicola* and a few cases of *C. theobromicola* (Chung *et al*., 2020; Zhang *et al*., 2020; Ji *et al*., 2022). Host‐associated clustering has been inconsistently reported for *C. nymphaeae* (Damm *et al*., 2012, Baroncelli *et al*., 2015), but our identification of a lineage (II) composed exclusively of non‐strawberry isolates suggests some host specialisation.

The clonal population structure and absence of a sexual morph indicate that *C. nymphaeae* is asexual (Damm *et al*., 2012). This is further supported by the absence of a a‐factor and incomplete α‐factor pheromone system in this species, as shown before by Wilson *et al*. (2021) for a single isolate. Within the CaSC, a sexual morph has only been observed for *C. salicis* and *C. rhombiforme*, both belonging to clade 5 (Damm *et al*., 2012). Consistent with this, all clade 5 species screened here possess a complete α‐pheromone/receptor pair, whereas the other CaSC species completely miss the α‐pheromone system or lack the receptor. Although the function of mating pheromones is unknown in *Colletotrichum* (Wilson *et al*., 2021), the presence of a complete α‐pheromone system may serve as an indicator of sexual reproduction in the CaSC. The α‐pheromone system was present in all screened CgSC species, but sexual reproduction for these species is not certain. The sexual morph has been observed for *C. fructicola* (Liang *et al*., 2021) and *C. gloeosporioides (*Weir *et al*., 2012), and recombining population structures have been reported for *C. siamense* and *C. horii*, but other species such as *C. kahawae* exhibit a clonal population structure (Vieira *et al*., 2018). This indicates that additional factors might be needed to predict sexual reproduction in the CgSC.

Most *C. nymphaeae* isolates belong to a single widespread clonal lineage (III‐B), consistent with earlier studies (Baroncelli *et al*., 2015, Wang *et al*., 2019). Global spread of this lineage likely occurred through the trade in infected runners and other planting material (Freeman, 2008). Despite the clonal genetic structure, virulence assays showed no lineage‐ or species‐specific differences, with most isolates capable of causing disease, consistent with previous findings (Baroncelli *et al*., 2015). This suggests that strawberry fruit pathogenicity is broadly conserved across *Colletotrichum* species, although confirmation on whole plants is needed. Nonetheless, and despite its apparent clonality, isolates of *C. nymphaeae* exhibited substantial variation in colony morphology and virulence in our assays, both within and among lineages. This pronounced phenotypic heterogeneity indicates ongoing diversification in *C. nymphaeae*, likely driven by mechanisms largely independent of sexual recombination.

Our analyses highlighted the contributions of multiple diversification mechanisms that do not rely on sexual recombination, including TE activity, the horizontal transfer of large starship elements among genomes, and variability in ACs. We now evaluate each mechanism in turn. TE content varies markedly among and within lineages, and TE insertions exhibit extensive presence/absence polymorphism, revealing a highly dynamic TE landscape within the species. The role of TEs in intraspecific diversity and fungal adaptation is becoming increasingly more evident (Faino *et al*., 2016; Oggenfuss *et al*., 2021; Gourlie *et al*., 2022; Nakamoto *et al*., 2023). Across numerous fungal taxa, TE content is a major driver of genome plasticity and is strongly correlated with genome size (Badet *et al*., 2020; Gan *et al*., 2021; Alkemade *et al*., 2024), a pattern we also observed in this study for *C. nymphaeae* and across members of the *Ca*SC and *Cg*SC. LTR/Ty3 elements contribute most to the observed genome size variation, which is also observed for other major fungal plant pathogens characterised by clonal lineages (Frantzeskakis *et al*., 2018; Rao *et al*., 2018; Oggenfuss *et al*., 2021; Stalder *et al*., 2023). In contrast to the related asexual *C. lupini* (Alkemade *et al*., 2024), LTR/Ty1 elements only represent a small fraction of the *C. nymphaeae* genome. LTR transposons have been shown to be de‐repressed under stress conditions and host colonisation (Fouché *et al*., 2020; Torres *et al*., 2021; Gupta *et al*., 2023; Alkemade *et al*., 2024) and to directly contribute to virulence in *Botrytis cinerea* (Porquier *et al*., 2021). Similarly, we detected de‐repression of LTR transposons during strawberry plant infection by *C. nymphaeae* (Ozbudak *et al*., 2025), suggesting a role for these elements in pathogenicity. Consistent with the ‘two‐speed’ genome theory (Dong *et al*., 2015; Torres *et al*., 2020), TEs were shown to cluster with accessory regions in the genome, highlighting their role in adaptation.

Starship elements seem to play a particularly important role in the diversification of *Colletotrichum*, as increasingly found in fungal plant pathogens (Gluck‐Thaler *et al*., 2022; Sato *et al*., 2025). Across the *Colletotrichum* genomes analysed in this study, we identified 24 distinct *Starship* haplotypes among 35 genomes, a number comparable to that reported for *Verticillium* species (Sato *et al*., 2025) and substantially higher than observed in *Aspergillus fumigatus* (Gluck‐Thaler *et al*., 2025). A unique *Prometheus Starship* was detected in a lineage III‐A isolate and contained genes commonly associated with virulence and stress responses, consistent with previously reported cases of *Starship*‐mediated transfer of virulence factors (Peck *et al*., 2024; Bucknell *et al*., 2025; Sato *et al*., 2025). In addition, two isolates from clonal lineage III‐A and one from III‐B shared identical *Phoenix Starship* elements, with homologous elements identified in the fellow clonal *Ca*SC member *C. lupini*. These *Phoenix Starships* were highly enriched in TEs and, aside from the conserved captain gene, only contained a single annotated cargo gene. Whether these *Starship* TEs are transcriptionally active remains to be determined, but such activity would be consistent with the hypothesis that active TEs enter fungal genomes via *Starship*‐mediated horizontal transfer (Sato *et al*., 2025; Griem‐Krey *et al*., 2026). Notably, both the presence and length of the identified *Starships* varied within clonal lineages, suggesting that they follow distinct evolutionary trajectories involving horizontal transfer rather than vertical inheritance. Furthermore, the *Phoenix Starship* contained SNP variation that changed significantly over time. A similar observation was recently reported in the asexual pathogen *V. nonalfalfae*, where temporal genomic change was significantly associated with an *Arwing Starship* element (Alkemade *et al*., 2025b). It should be investigated if temporal change is more often associated with *Starship* elements, and if this is more common in clonal species compared to sexually recombining ones. Within the *Starship*, the identified SNPs were all located within the same unclassified TE, which shares homologs with *C. lupini* that are also located within a *Phoenix Starships*, further suggesting a role for horizontal transfer in shaping genomic variation.

As the third major category of asexual diversification mechanisms, we found that *C. nymphaeae* isolates also vary in the presence or absence of ACs. Many *Colletotrichum* species, such as *C. graminicola*, *C. higginsianum* and members of the *Cg*SC, have 10 core chromosomes and a variable number of ACs enriched in virulence and horizontally transferred genes (Plaumann *et al*., 2018; Becerra *et al*., 2023; Wang *et al*., 2023). Within the *Ca*SC, *C. lupini* also showed 10 core chromosomes and one AC with an unclear function (Alkemade *et al*., 2024), but *C. scovilei*, closely related to *C. nymphaeae*, showed 14 core chromosomes and 1 accessory (Wang *et al*., 2023). By contrast, we show that *C. nymphaeae* consists of 11 or 13 core chromosomes, with a variable number of TE‐rich mini ACs/contigs. Both Cnym01 AC 14 and accessory contig 15 are rich in gene duplications, suggesting a major role in AR evolution as suggested by (van Westerhoven *et al*., 2024). While differences in chromosome number or large‐scale duplications can be associated with fitness costs (Todd *et al*., 2017), they can also generate genetic variation that facilitates adaptation, such as enhanced virulence or fungicide resistance (Sionov *et al*., 2010; Ropars *et al*., 2018). AC 14 of Cnym01 (I), contains stress and detoxification‐related genes such as Zn2Cys6 transcription factors and MFS transporters (Lin *et al*., 2018; Vela‐Corcía *et al*., 2019; Lu *et al*., 2023; John *et al*., 2024; Yang *et al*., 2024). This AC also contained a duplication of a cytochrome P450, which are the primary targets for azole‐based fungicides (Yoshida, 1988), and could be involved in observed fungicide resistance within *C. nymphaeae* (Usman *et al*., 2022). Similar to AC 14 of Cnym01, the accessory contig 14 observed within lineage III‐B, which was highly upregulated during leaf and fruit infection, also contained a unique cytochrome P450, and Zn2Cys6 encoding genes. This contig also contained a 2 kb region that is conserved among members of the *Cg*SC but absent from the *Ca*SC and harbours two predicted effector genes, strongly suggesting that the region was horizontally transferred from the *Cg*SC into lineage III‐B. Notably, this region was flanked by DNA/TcMar elements, further supporting a potential role for this type of transposon in facilitating HGT (Romeijn *et al*., 2025). Overall, the observed variability in ACs, partly driven by horizontal transfer events, further highlights their contribution to genomic diversification, as has been reported for other clonal fungal plant pathogens (Akagi *et al*., 2009; Barragan *et al*., 2024; Peck *et al*., 2024).

Collectively, the three reported mechanisms of asexual diversification contribute to variability in pangenome content in *C. nymphaeae*. With *c*. 74% of genes shared among all isolates, indicating a largely stable core genome comparable to other *Colletotrichum* species (Becerra *et al*., 2023; Alkemade *et al*., 2024), although broader sampling across hosts may further refine this estimate. Accessory genes were less conserved and evolved under relaxed selective pressure compared to core genes and effectors, consistent with patterns observed in other fungal plant pathogens (Alkemade *et al*., 2024; van Westerhoven *et al*., 2024). Gene duplication appears to be a major source of accessory genome diversification (van Westerhoven *et al*., 2024), as duplicated genes were enriched in ARs and mini‐chromosomes of the reference genome Cnym01. The clustering of ARs with clonal lineages supports a model in which rare or ancient sexual recombination is followed by clonal expansion, during which meiotic irregularities may generate copy number variation and AR diversification. Subsequent clonal propagation would preserve lineage‐specific AR architectures, explaining their similarity within lineages and divergence between them. Similar dynamics have been described in *Zymoseptoria tritici*, where chromosomal duplication, breakage and fusion shape accessory genome evolution (Croll & McDonald, 2012; Möller & Stukenbrock, 2017). In addition, the enrichment of TEs in ARs likely further promotes structural variation through duplications, rearrangements and HGT (Faino *et al*., 2016; Torres *et al*., 2021; Stalder *et al*., 2023).

To conclude, we showed that strawberry anthracnose in Europe and North America is mainly caused by *C. nymphaeae*, largely represented by a single, widely spread clonal lineage. No clear difference in strawberry fruit virulence between *C. nymphaeae* lineages and other strawberry‐infecting *Colletotrichum* species was observed, indicating that strawberry pathogenicity is widespread within *Colletotrichum*. Despite clonality and the apparent absence of sexual recombination, considerable phenotypic and genomic variation exists within *C. nymphaeae*. This diversity is primarily driven by TE‐rich ARs and *Starship* elements. ARs are largely lineage‐specific, highly dynamic and enriched for gene duplications, horizontally transferred genes and genes likely involved in adaptation and virulence. Transcriptional activation of accessory genes, effectors and TEs during infection further highlights their functional relevance. Our findings show that ARs are associated with diversification and adaptation in *C. nymphaeae*, which can aid in the identification of pathogenicity genes to support disease management and resistance gene discovery in strawberry.

## Competing interests

None declared.

## Author contributions

JAA collected data, performed the analyses and wrote the manuscript. AGB and AK maintained and retrieved the CABI isolates. TGB conceived and supervised the project. All authors contributed to writing and editing.

## Disclaimer

The New Phytologist Foundation remains neutral with regard to jurisdictional claims in maps and in any institutional affiliations.

## Supporting information


**Fig. S1** Genome statistics *Colletotrichum* species and *C. nymphaeae* lineages.
**Fig. S2** Principal component analysis based on 42 715 SNPs, dividing *C. nymphaeae* into five distinct lineages.
**Fig. S3** *Colletotrichum nymphaeae* cluster analysis based on 42 715 SNPs.
**Fig S4** Collection points of *Colletotrichum nymphaeae* isolates and strawberry production in tonnes.
**Fig. S5** MAT1 locus and mating‐type pheromone systems in *Colletotrichum*.
**Fig. S6** Correlations between virulence and growth rate.
**Fig. S7** *Colletotrichum nymphaeae* isolate morphology.
**Fig. S8** Transposable element (TE) landscape of members of the *Colletotrichum gloeosporioides* and *acutatum* species complex.
**Fig. S9** Pearson correlations between *Colletotrichum* assembly statistics and virulence vs transposable element content (TE) and genome size (Mb).
**Fig. S10** Mean TE subclass length in bp.
**Fig. S11** Average length of transposable element subclasses across *Colletotrichum nymphaeae* lineages.
**Fig. S12** TE divergence (kimura 2‐parameter distance) landscapes across *Colletotrichum nymphaeae* genomes.
**Fig. S13** Temporal analysis of *Colletotrichum nymphaeae* lineage III‐B.
**Fig. S14** Phylogeny of unclassified element (MYCMOB1.1_FAMILY‐174 522‐COLLUP_NE_1662) located within *Phoenix Starship* (Ph1h1) of *Colletotrichum nymphaeae* isolate Cnym02.
**Fig. S15** Synteny of *Colletotrichum nymphaeae* long‐read assemblies and accessory chromosomes/contigs.
**Fig. S16** Genome compartmentalisation and accessory regions (ARs) in *Colletotrichum nymphae*a isolates.
**Fig. S17** Clustering based on accessory orthologous group (OG) gene count of *Colletotrichum nymphaeae*.
**Fig. S18** De‐repression of transposable element (TE) superfamilies during strawberry leaf and fruit infection.
**Fig. S19** Expression of gene categories during strawberry leaf and fruit infection.
**Fig. S20** Expression per chromosome during strawberry leaf and fruit infection.
**Fig. S21** Clustering based on effector orthologous group (OG) gene count of *Colletotrichum*.
**Fig. S22** Gene trees of OG0603 and OG0604.

**Table S1** Isolate and genome details.
**Table S2** Transcriptomic data used in this study.
**Table S3** Lineage and population diversity statistics.
**Table S4** *Colletotrichum Starships* identified in this study.
**Table S5** Functional information of predicted species/lineage‐specific effectors and accessory region‐related genes.
**Table S6** Variants associated with temporal change and their associated candidate genes, TEs and protein sequences.
Please note: Wiley is not responsible for the content or functionality of any Supporting Information supplied by the authors. Any queries (other than missing material) should be directed to the *New Phytologist* Central Office.

## Data Availability

The data that support the findings of this study are openly available in the Sequence Read Archive at https://www.ncbi.nlm.nih.gov/sra. The data sequenced in this study are available under accession no. PRJNA1376502. All individual accession numbers from this study and other public sources are listed in Table S1.
