# Supplementary material for: Accessory regions and horizontal gene transfer shape the evolution of clonal Colletotrichum nymphaeae infecting strawberry
Source: New Phytol. 2026 Jun 5;251(4):2186–205. doi: 10.1111/nph.71314 (PMC13373834; doi:10.1111/nph.71314)
Supplement: Supplementary file 1 — Fig. S1 Genome statistics Colletotrichum species and C. nymphaeae lineages. Fig. S2 Principal component analysis based on 42 715 SNPs, dividing C. nymphaeae into five distinct lineages. Fig. S3 Colletotrichum nymphaeae cluster analysis based on 42 715 SNPs. Fig S4 Collection points of Colletotrichum nymphaeae isolates and strawberry production in tonnes. Fig. S5 MAT1 locus and mating‐type pheromone systems in Colletotrichum. Fig. S6 Correlations between virulence and growth rate. Fig. S7 Colletotrichum nymphaeae isolate morphology. Fig. S8 Transposable element (TE) landscape of members of the Colletotrichum gloeosporioides and acutatum species complex. Fig. S9 Pearson correlations between Colletotrichum assembly statistics and virulence vs transposable element content (TE) and genome size (Mb). Fig. S10 Mean TE subclass length in bp. Fig. S11 Average length of transposable element subclasses across Colletotrichum nymphaeae lineages. Fig. S12 TE divergence (kimura 2‐parameter distance) landscapes across Colletotrichum nymphaeae genomes. Fig. S13 Temporal analysis of Colletotrichum nymphaeae lineage III‐B. Fig. S14 Phylogeny of unclassified element (MYCMOB1.1_FAMILY‐174 522‐COLLUP_NE_1662) located within Phoenix Starship (Ph1h1) of Colletotrichum nymphaeae isolate Cnym02. Fig. S15 Synteny of Colletotrichum nymphaeae long‐read assemblies and accessory chromosomes/contigs. Fig. S16 Genome compartmentalisation and accessory regions (ARs) in Colletotrichum nymphaea isolates. Fig. S17 Clustering based on accessory orthologous group (OG) gene count of Colletotrichum nymphaeae. Fig. S18 De‐repression of transposable element (TE) superfamilies during strawberry leaf and fruit infection. Fig. S19 Expression of gene categories during strawberry leaf and fruit infection. Fig. S20 Expression per chromosome during strawberry leaf and fruit infection. Fig. S21 Clustering based on effector orthologous group (OG) gene count of Colletotrichum. Fig. S22 Gene trees of OG0603 and OG0604. [file NPH-251-2186-s001.docx]

## *New Phytologist* Supporting Information

Article title: **Accessory regions and horizontal gene transfer shape evolution of clonal *Colletotrichum nymphaeae* infecting strawberry**

Authors: **Joris A. Alkemade, Alan G. Buddie, Anthony Kermode, Timothy G. Barraclough**

Article acceptance date: 15 May 2026

The following Supporting Information is available for this article:

**Fig. S1** **Genome statistics *Colletotrichum* species and *C. nymphaeae* lineages.**

**Fig. S2** **Principal component analysis based on 42,715 SNPs, dividing *C. nymphaeae* in 5 distinct lineages.**

**Fig. S3** ***Colletotrichum nymphaeae* cluster analysis based on 42,715 SNPs.**

**Fig. S4** **Collection points of *Colletotrichum nymphaeae* isolates and strawberry production in tonnes.**

**Fig. S5** **MAT1 locus and mating-type pheromone systems in *Colletotrichum*.**

**Fig. S6** **Correlations between virulence and growth rate.**

**Fig. S7** ***Colletotrichum nymphaeae* isolate morphology.**

**Fig. S8** **Transposable element (TE) landscape of members of the *Colletotrichum gloeosporioides* and *acutatum* species complex.**

**Fig. S9** **Pearson correlations between *Colletotrichum* assembly statistics and virulence versus transposable element content (TE) and genome size (Mb).**

**Fig. S10** **Mean TE subclass length in bp.**

**Fig. S11** **Average length of transposable element subclasses across *Colletotrichum nymphaeae* lineages.**

**Fig. S12** **TE divergence (kimura 2-parameter distance) landscapes across *Colletotrichum* *nymphaeae* genomes.**

**Fig. S13** **Temporal analysis of *Colletotrichum nymphaeae* lineage III-B*.***

**Fig. S14** **Phylogeny of unclassified element (MYCMOB1.1_FAMILY-174522-COLLUP_NE_1662) located within *Phoenix Starship* (Ph1h1) of *Colletotrichum nymphaeae* isolate Cnym02.**

**Fig. S15** **Synteny of *Colletotrichum nymphaeae* long-read assemblies and accessory chromosomes / contigs.**

**Fig. S16** **Genome compartmentalization and accessory regions (ARs) in *Colletotrichum nymphae*a isolates.**

**Fig. S17** **Clustering based on accessory orthologous group (OG) gene count of *Colletotrichum nymphaeae*.**

**Fig. S18** **De-repression of transposable element (TE) superfamilies during strawberry leaf and fruit infection.**

**Fig. S19** **Expression of gene categories during strawberry leaf and fruit infection.**

**Fig. S20** **Expression per chromosome during strawberry leaf and fruit infection.**

**Fig. S21** **Clustering based on effector orthologous group (OG) gene count of *Colletotrichum*.**

**Fig. S22** **Gene trees of (a) OG0603 and (b) OG0604.**

**Table S1 Isolate and genome details.**

**Table S2 Transcriptomic data used in this study.**

**Table S3 Lineage and population diversity statistics.**

**Table S4 *Colletotrichum Starships* identified in this study.**

**Table S5 Functional information of predicted species/lineage specific effectors and accessory region related genes.**

**Table S6 Variants associated with temporal change and their associated candidate genes, TEs and protein sequences.**

**Fig. S1** **Genome statistics *Colletotrichum* species and *C. nymphaeae* lineages.** **(a – d)** Genome size (mb), GC content (%), number of genes and transposable element (TE) content of members of the C. g*loeosporioides* species complex (*Cg*SC), the *C. acutatum* species complex (*Ca*SC), clade II of the *Ca*SC and *C. nymphaeae,* **(e – h)** and of *C. nymphaeae* lineages I, II, III, III-A and III-B. Big black dot within boxplot indicates the mean.


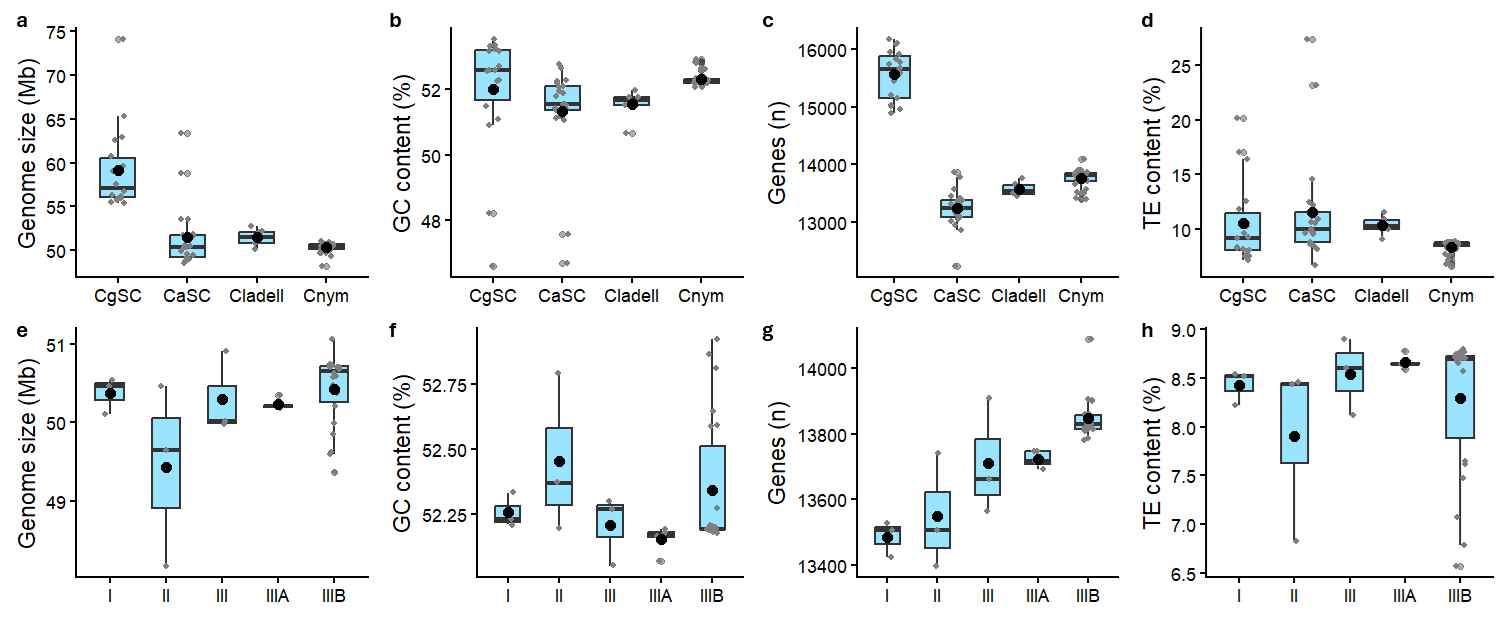


**Fig. S2** **Principal component analysis based on 42,715 SNPs, dividing *C. nymphaeae* in 5 distinct lineages.** PCA of 42,715 SNPs using PC1 and PC3. **(b)** PCA of PC2 and PC3.

**
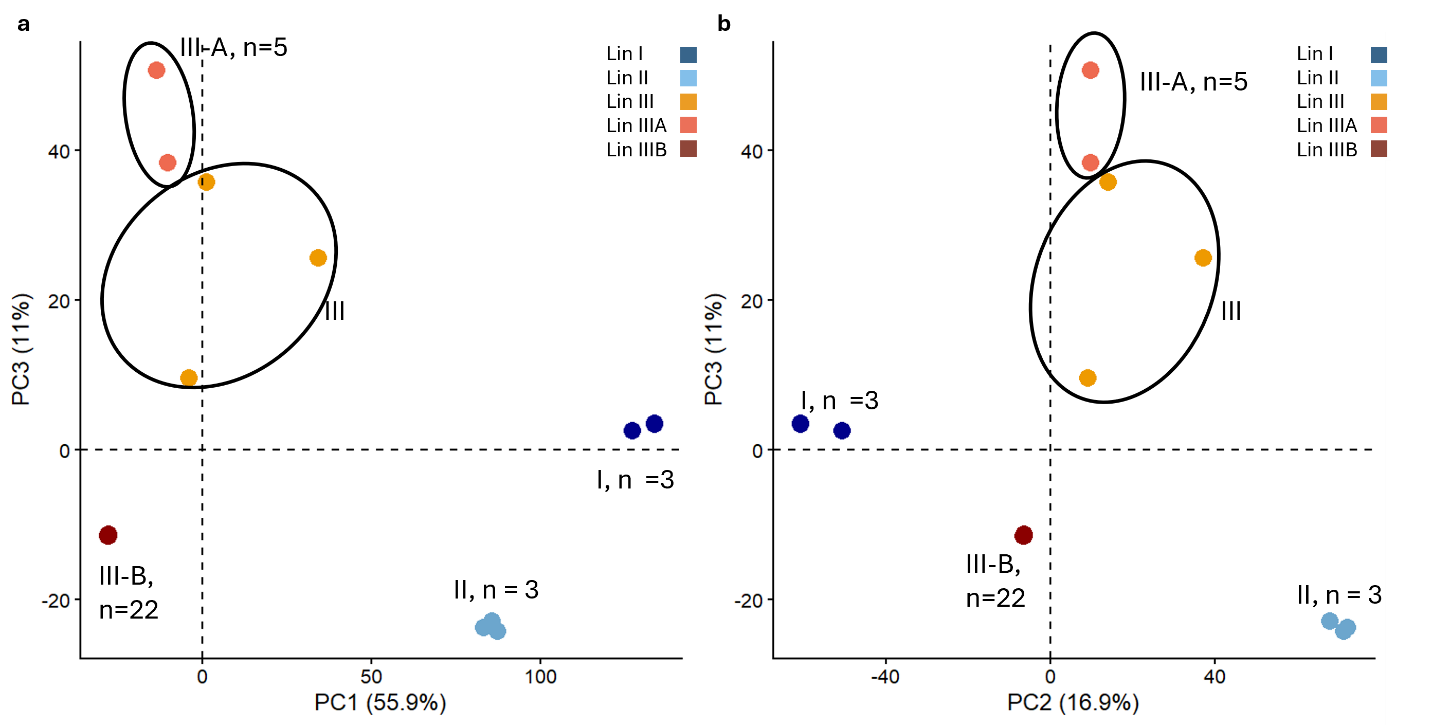
**

**Fig. S3** ***Colletotrichum nymphaeae* cluster analysis based on 42,715 SNPs. (a)** BIC model fit, **(b)** AIC model fit and **(c)** DAPC with 2 PCAs and 2 DAs retained.

**
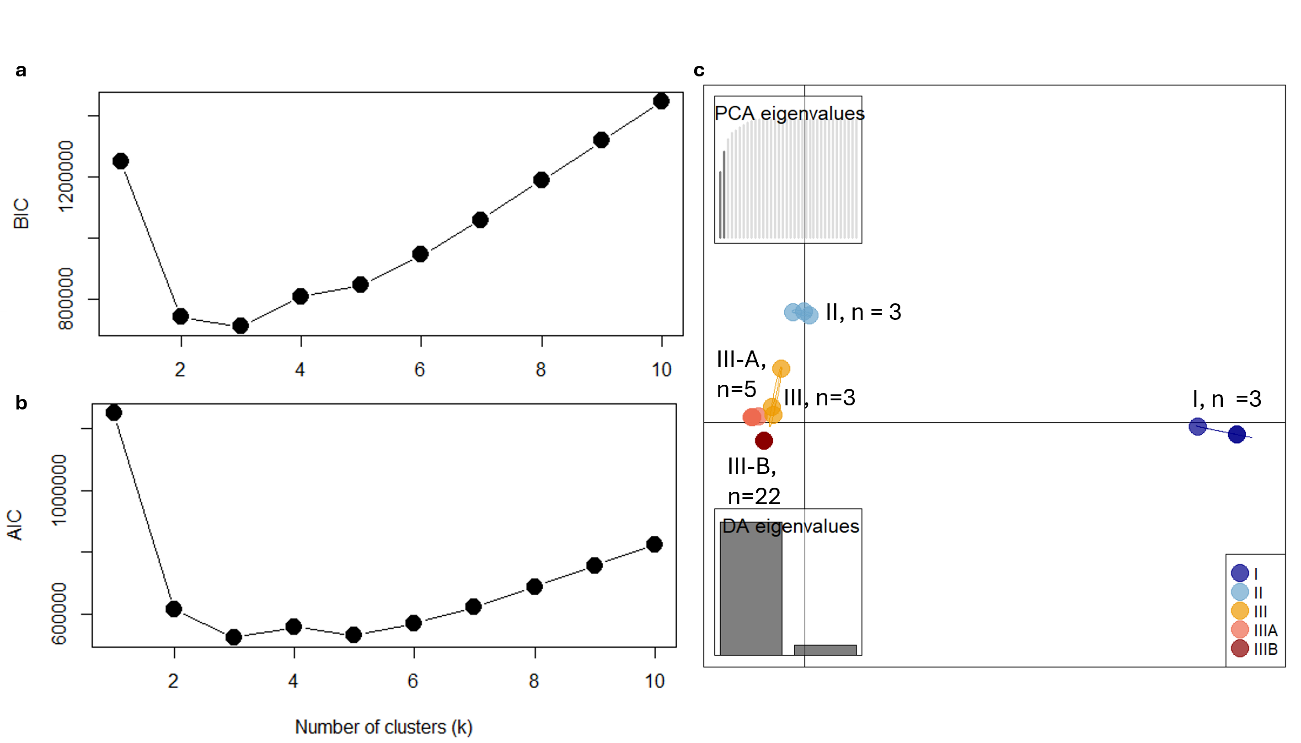
**

**Fig. S4** **Collection points of *Colletotrichum nymphaeae* isolates and strawberry production in tonnes.**


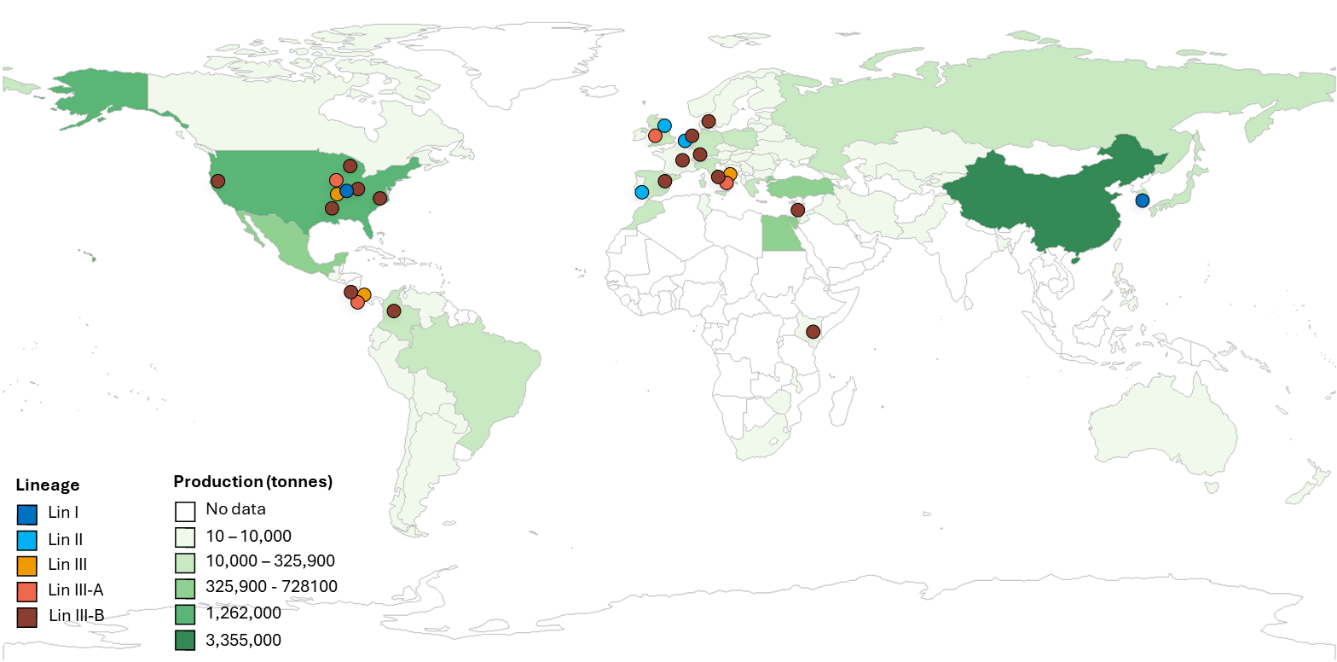


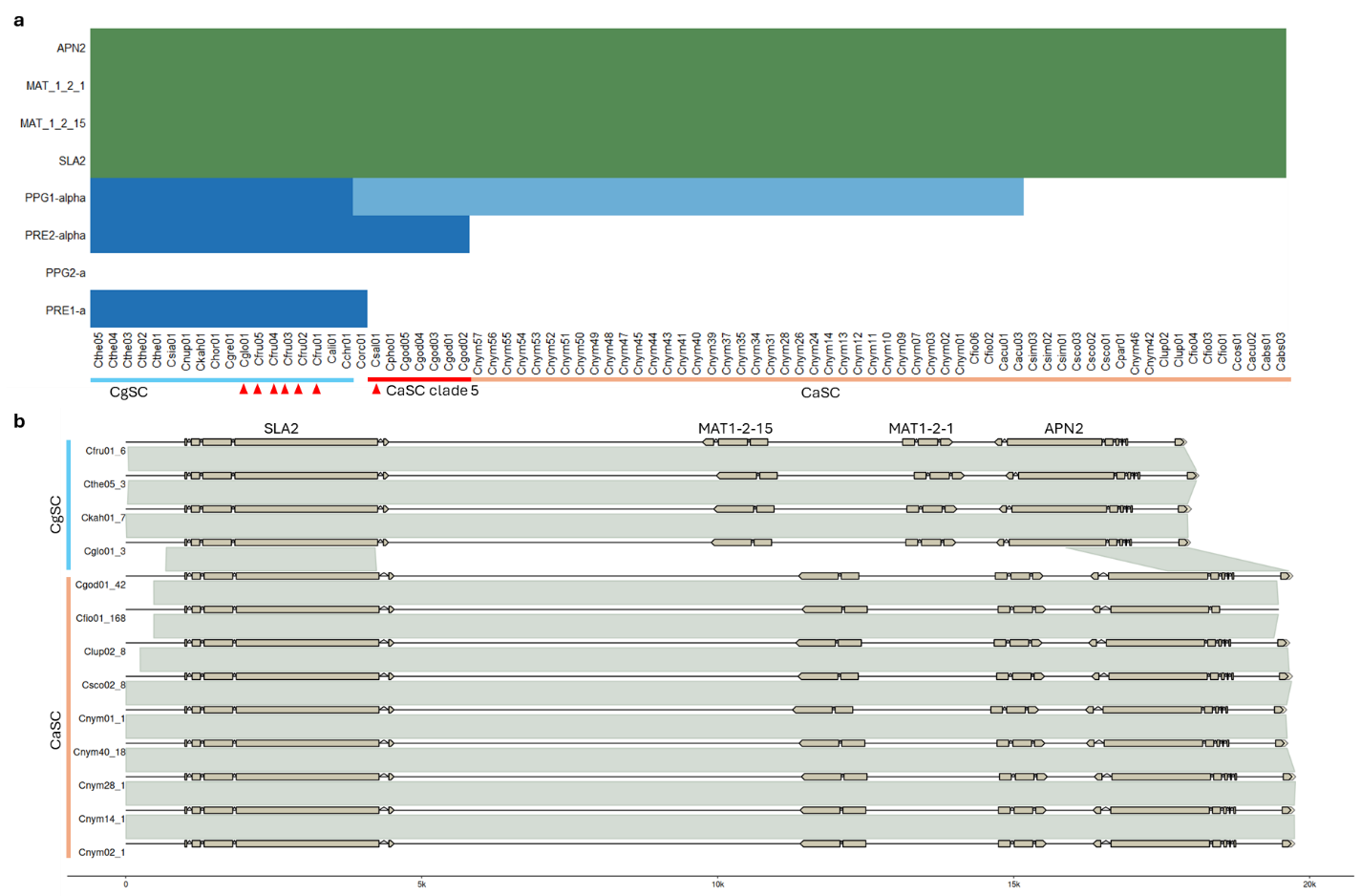
**Fig. S5** **MAT1 locus and mating-type pheromone systems in *Colletotrichum*. (a)** Presence (colored) and absence (white) matrix of MAT1 locus genes (green), along with mating-type pheromones (PPG1-α and PPG2-a) and their corresponding receptors (PRE2-α and PRE1-a; blue). Light blue denotes the *C. acutatum* species complex variant of PPG1-α. A red triangle indicates observed sexual morph. **(b)** The MAT1 locus exhibits high synteny within species complexes. However, aside from the flanking genes, the remainder of the locus is less conserved between different species complexes.

**Fig. S6** **Correlations between virulence and growth rate. (a)** Pearsons correlation (R) between virulence and growth of all tested *Colletotrichum* species. **(b)** Pearsons correlation (R) between virulence and growth of tested *Colletotrichum nymphaeae* isolates.


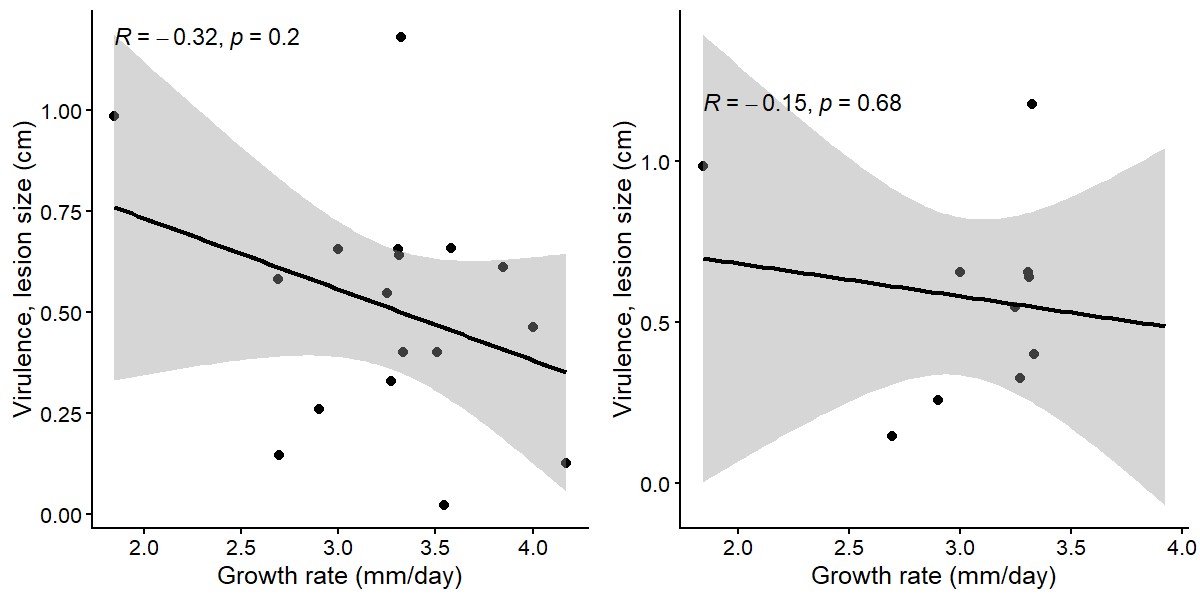


**Fig. S7** ***Colletotrichum nymphaeae* isolate morphology.** Isolate name and lineage followed by photo of front and reverse of petri dish containing 7 days old colony grown on PDA.


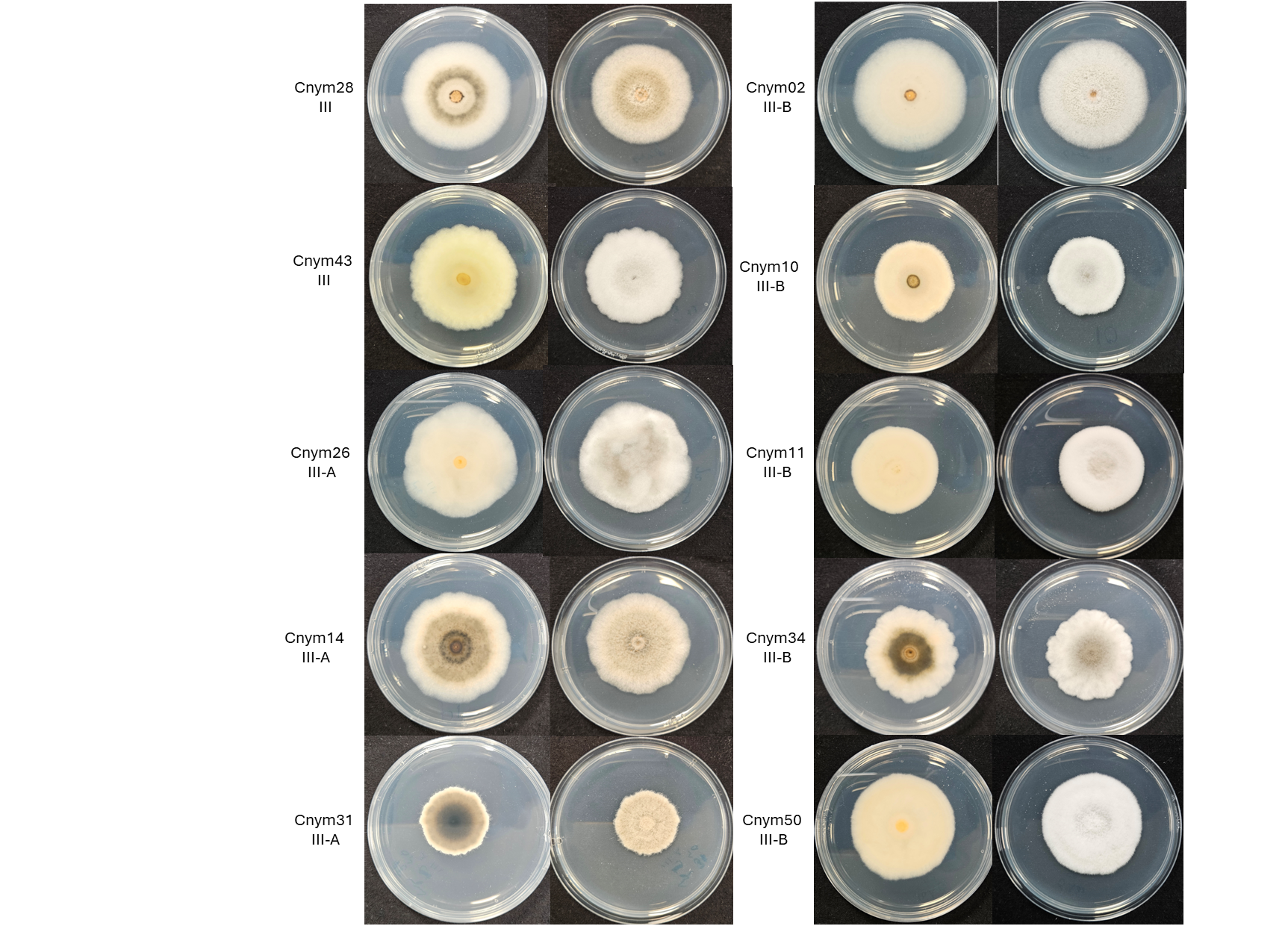


**Fig. S8** **Transposable element (TE) landscape of members of the *Colletotrichum gloeosporioides* and *acutatum* species complex. (a)** Contribution of TE superfamilies to genome size, legend is found below and bars on top indicate species or species complex (CaSC: *C. acutatum* species complex; CgSC: *C. gloeosporioides* complex). **(b)** Proportion of TE superfamilies across all genomes with 100% referring to the total TE content of the respective genomes. **(c)** Correlation (Pearson) of TE content (%) to genome size of all included species. **(d)** Correlation of TE content (%) to genome size of CaSC species. **(e)** TE divergence (kimura 2-parameter distance) landscapes across *Colletotrichum* genomes, with the y-axis showing genomic proportion (Mb) occupied by each TE class and the x-axis indicating K2P distance. Peaks represent bursts of TE expansion, where younger elements (<0.1) indicate recent activity while older elements (>0.1) reflect ancestral bursts. TE class colors are presented in **(f)**, showing TE divergence landscapes for each TE class separately.

**
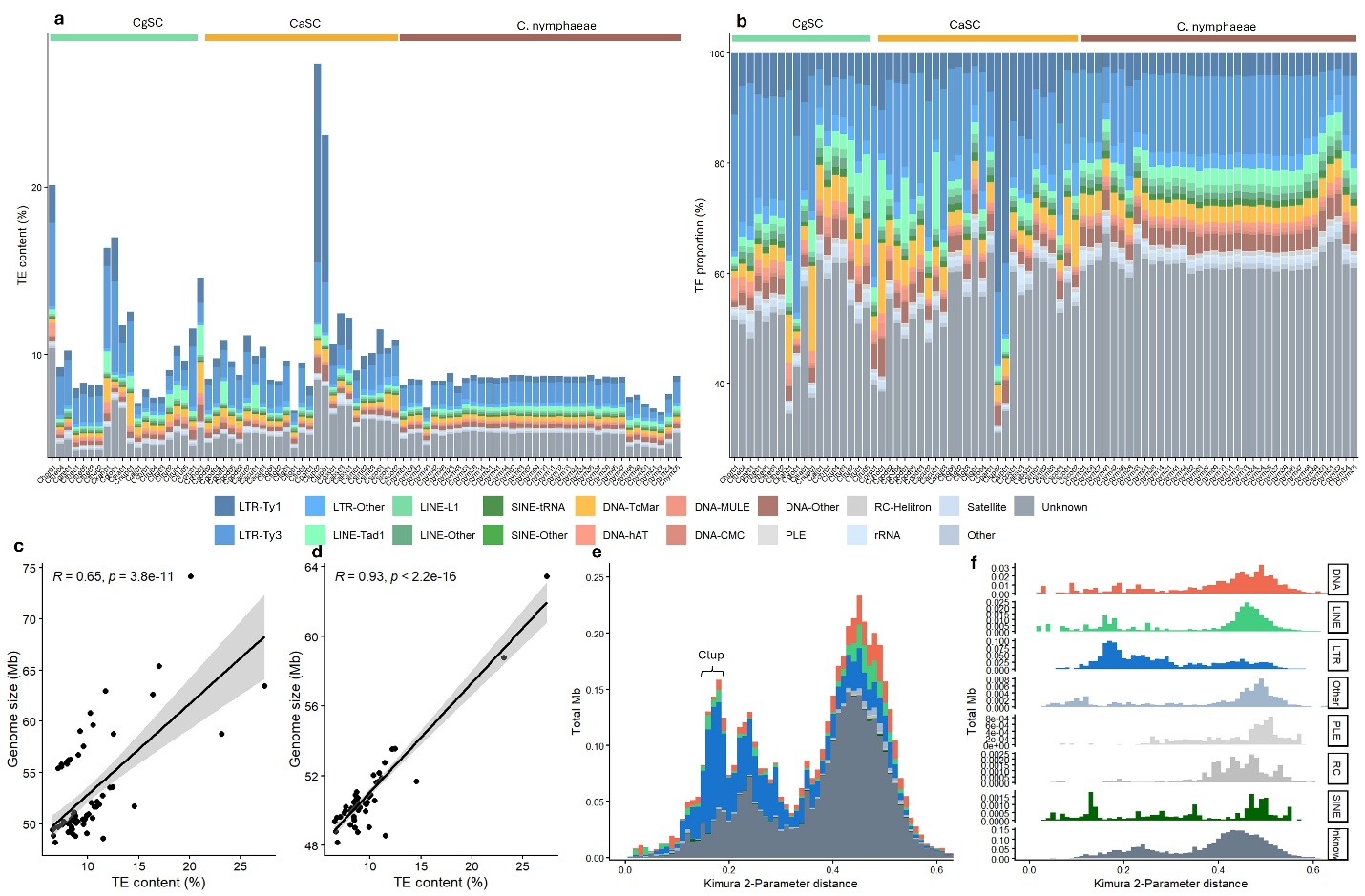
**

**Fig. S9** **Pearson correlations between *Colletotrichum* assembly statistics and virulence versus transposable element content (TE) and genome size (Mb).** First column: number of Ns per 100 kb in assembly. Second column: assembly coverage. Third column: assembly fragmentation in number of contigs. Fourth column: virulence on strawberry fruit in lesion size (cm). First and third row show correlations of all assemblies and the second and fourth row only show *C. nymphaeae* assemblies.

**
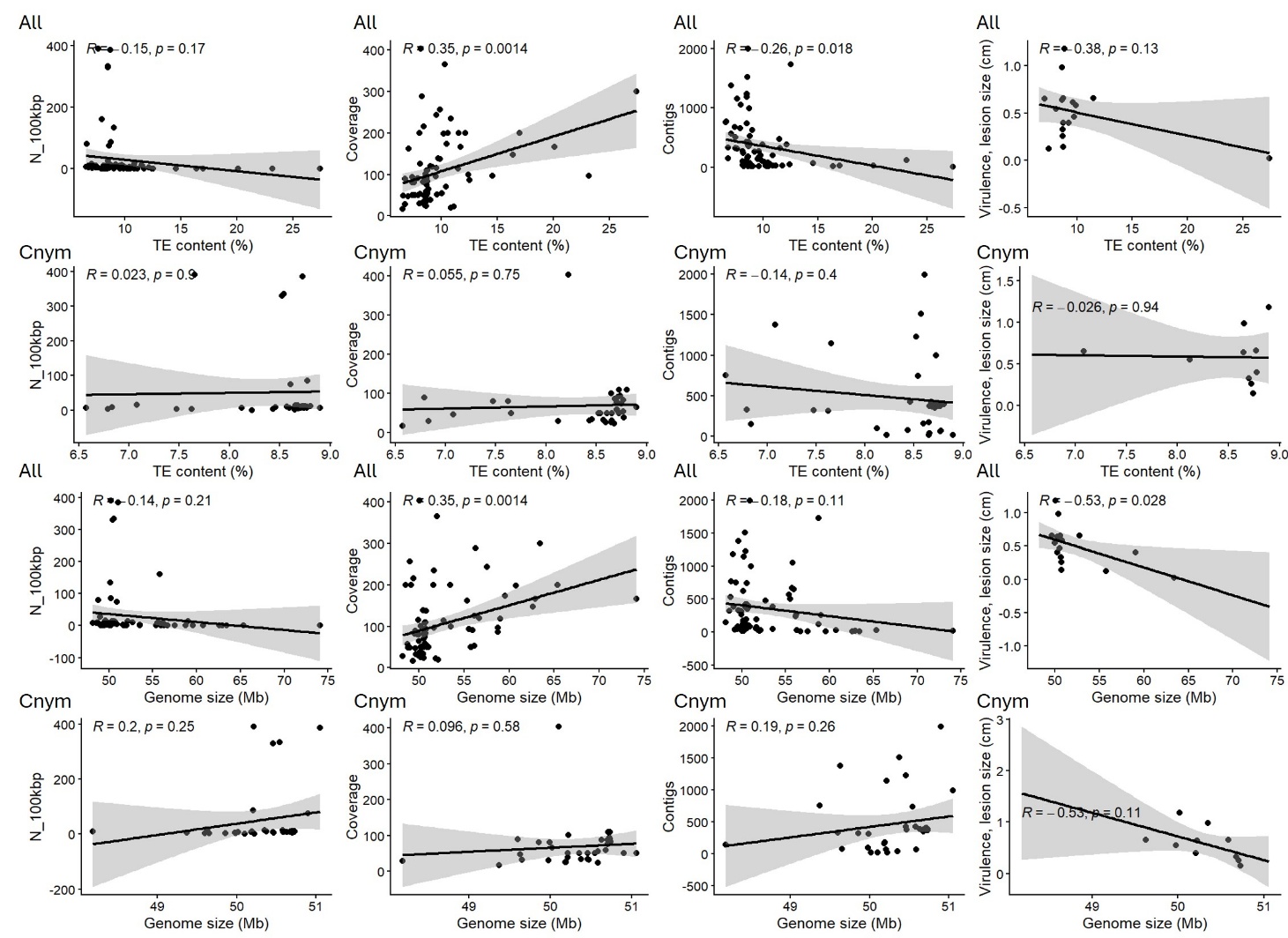
**

**Fig. S10** **Mean TE subclass length in bp. (a)** TE subclass length of all assessed *Colletotrichum* species. **(b)** TE subclass length of all assessed *C. nymphaeae* isolates. Big gray dot within boxplot indicates the mean.

**
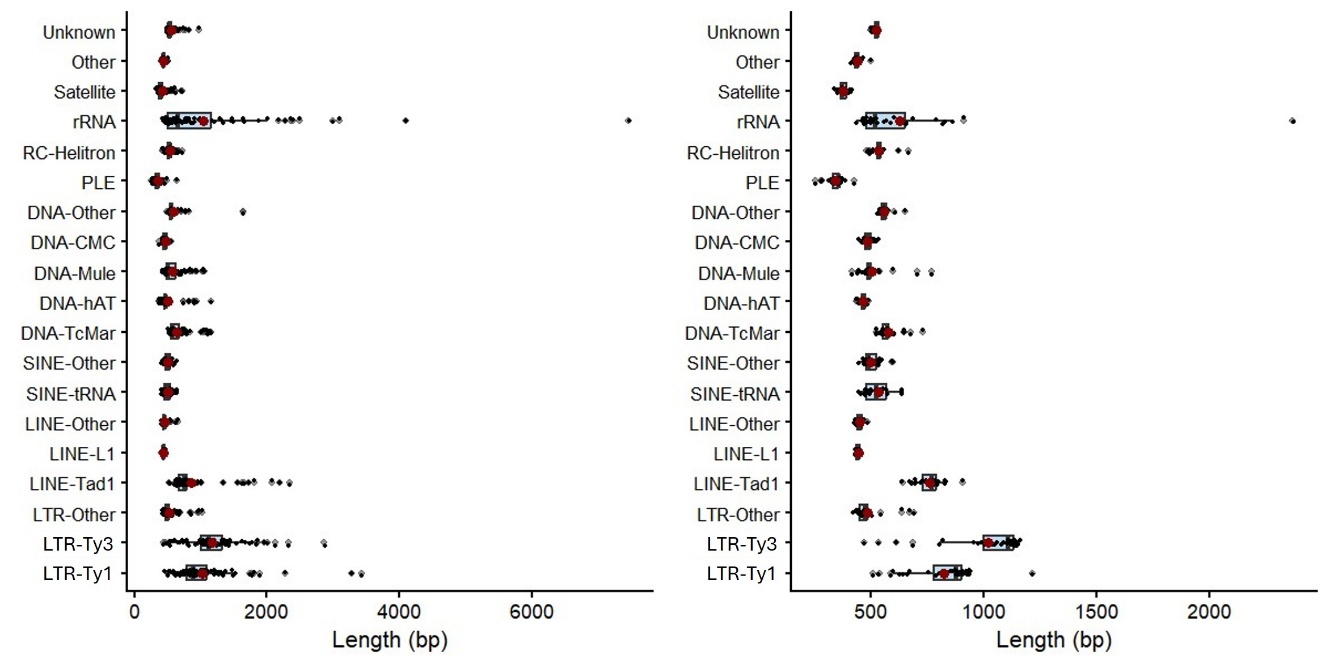
**

**Fig. S11** **Average length of transposable element subclasses across *Colletotrichum nymphaeae* lineages.** The y axis in length in bp of TE type and the x axis indicates *C. nymphaeae* lineage.

**
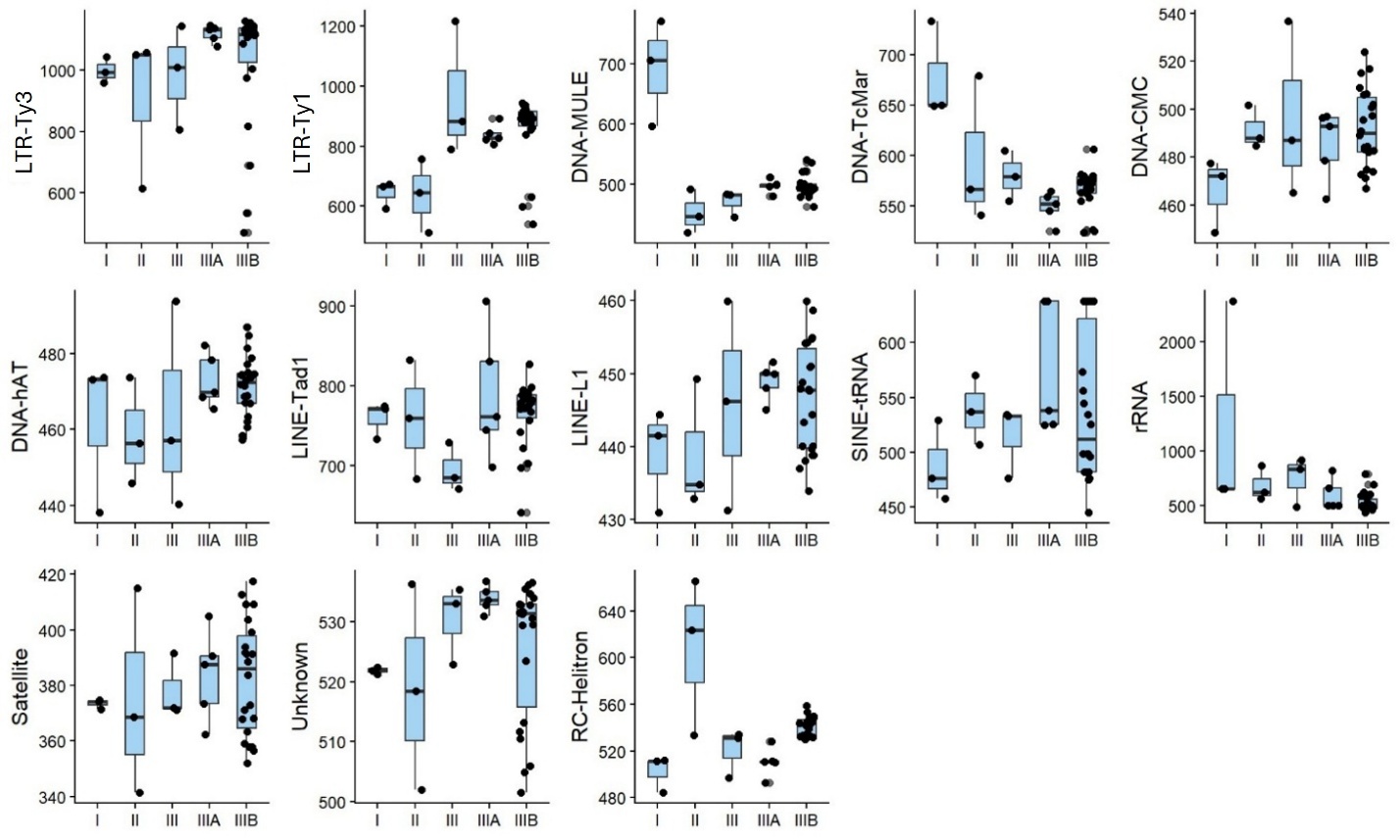
**

**Fig. S12** **TE divergence (kimura 2-parameter distance) landscapes across *Colletotrichum* *nymphaeae* genomes.** The y-axis shows genomic proportion (Mb) occupied by each TE class and the x-axis indicates K2P distance. Peaks represent bursts of TE expansion, where younger elements (<0.1) indicate recent activity while older elements (>0.1) reflect ancestral bursts.


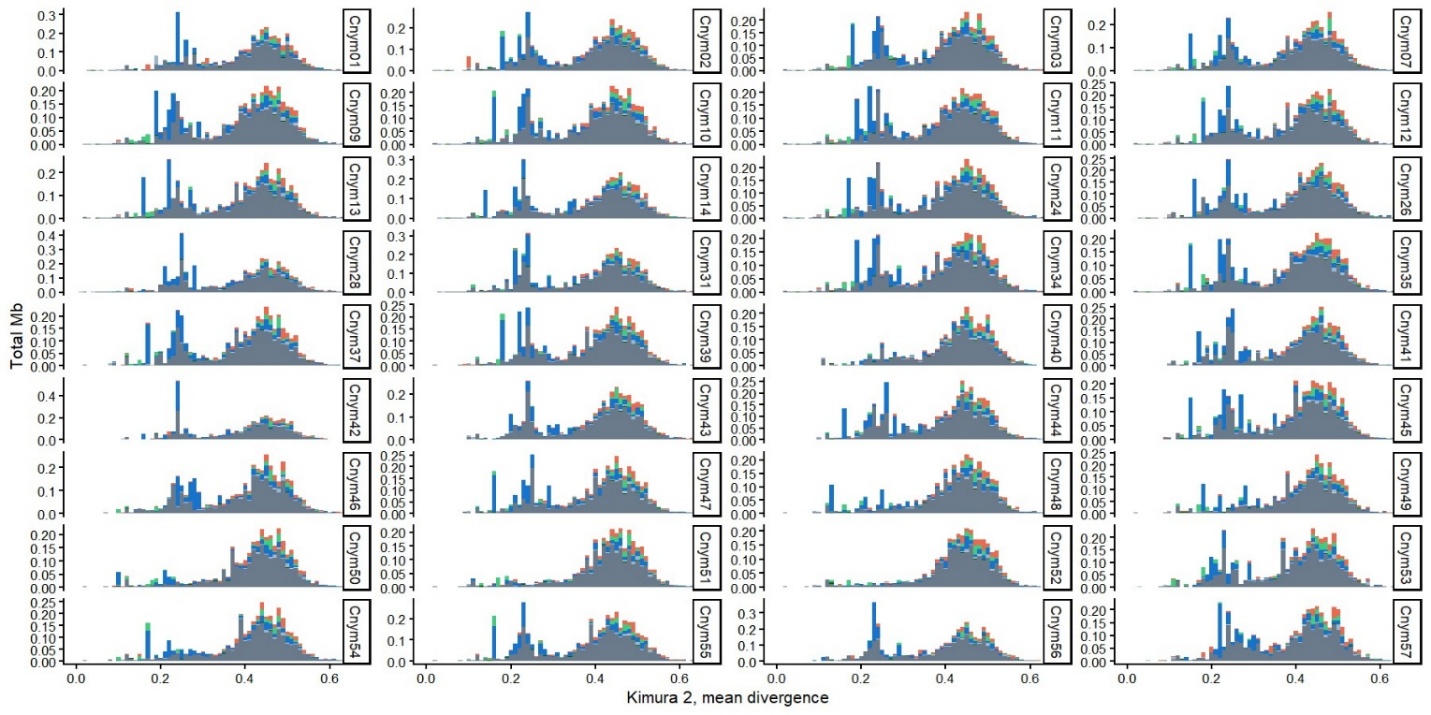


**Fig. S13** **Temporal analysis of *Colletotrichum nymphaeae* lineage III-B*.* (a)** Histogram of collection time points, **(b)** binomial regression plot (*P* < 0.01), **(c)** Boxplots showing allele change over time.

**
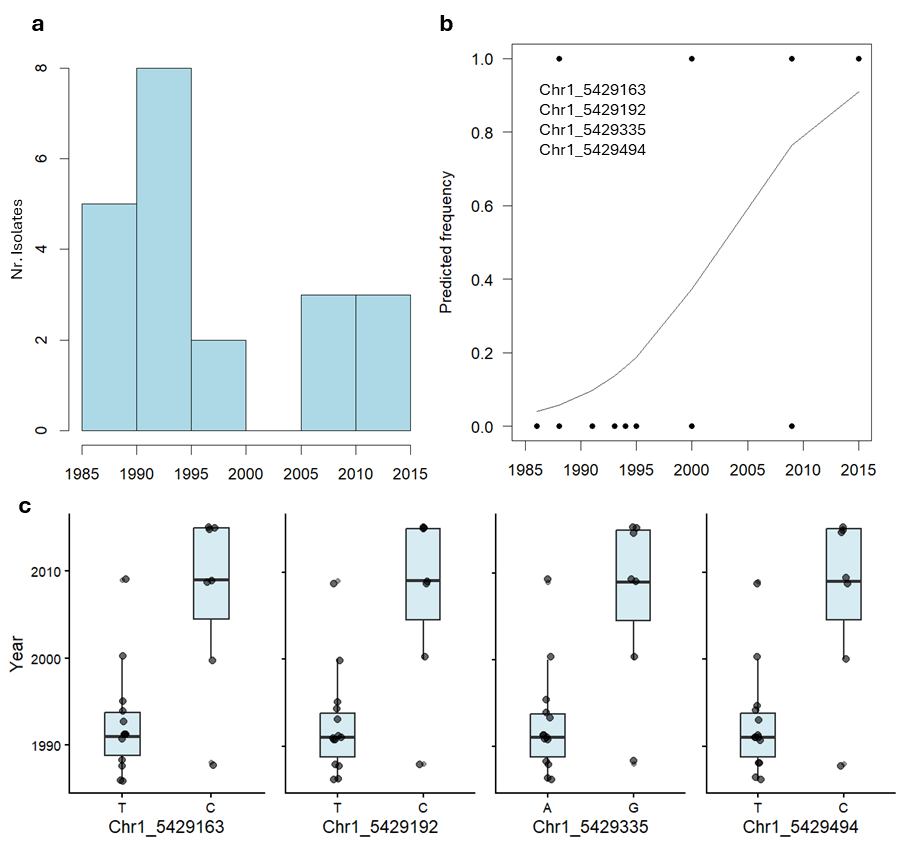
**

**Fig. S14** **Phylogeny of unclassified element (MYCMOB1.1_FAMILY-174522-COLLUP_NE_1662) located within *Phoenix Starship* (Ph1h1) of *Colletotrichum nymphaeae* isolate Cnym02.** The *Phoenix Starship* haplotypes are indicated in red. Phylogeny is based on k-mer similarity. Element homologs are only found in *C.* *nymphaeae lineage* III (A & B) isolates, *C. lupini* and *C. costaricence*.


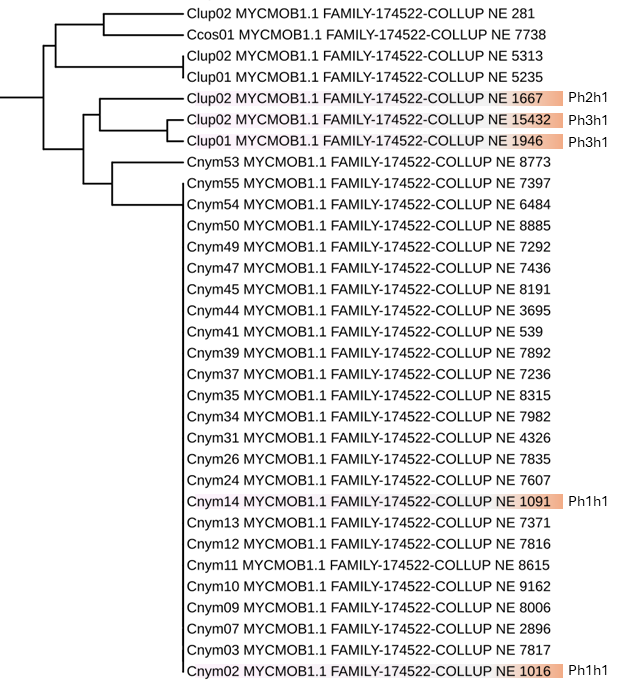


**Fig. S15** **Synteny of *Colletotrichum nymphaeae* long-read assemblies and accessory chromosomes / contigs.** Synteny between, in blue, Cnym01 (I) and in grey, **(a)** Cnym28 (III), **(b)** Cnym26 (III-A), **(c)** Cnym14 (III-A), **(d)** Cnym31 (III-A) and **(e)** Cnym02 (III-B) compared to Cnym01 (I). **(f)** Accessory content per chromosome / contig (> 50 kb) per assembly.


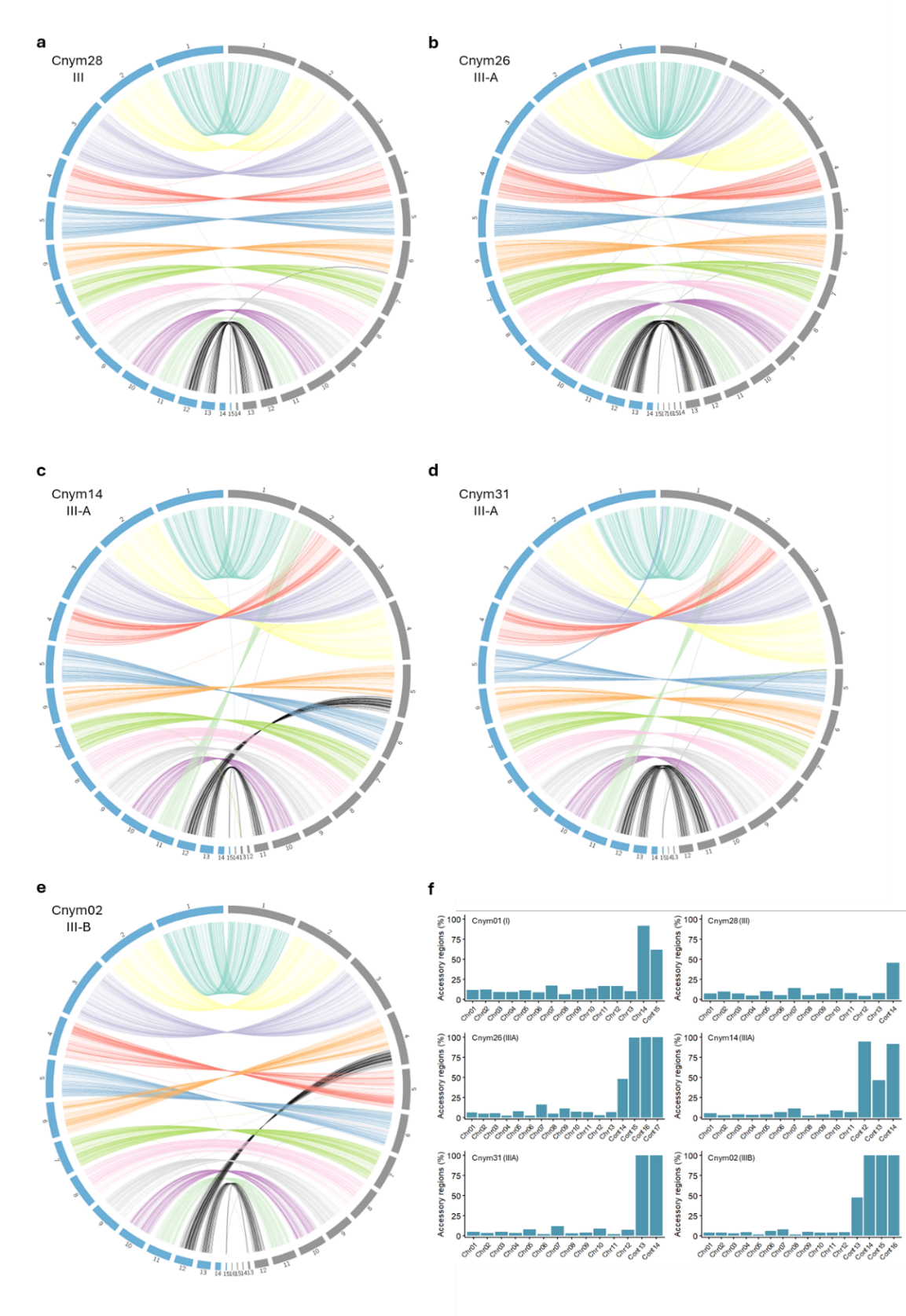


**Fig. S16** **Genome compartmentalization and accessory regions (ARs) in *Colletotrichum nymphae*a isolates. (a)** Genome overview of Cnym02, showing ARs (light green) are clustered together with transposable elements (TEs; bottom red line) rich and gene (upper blue line) poor regions. A similar pattern is found for the sub-telomeric regions (grey, first and last 5% of the chromosome). Red line in Chr1 indicates *Starship* Ph1H1 **(b)** This pattern is further accentuated by principal component analysis (PCA) on gene-, TE-, GC-content and coverage of 36 *C. nymphaeae* strains, clustering ARs (light blue) and sub-telomeric regions with high TE density and core (dark red) and softcore (red) regions with high gene density and coverage. **(c)** Accessory contigs (gray line), both contigs are gene poor (blue density plot) and TE rich (black). **(d)** Genome overview of Cnym14, red line in Chr1 indicates *Starship* Ph1H1. **(e)** Genome feature PCA on gene-, TE-, GC-content and coverage and **(f)** accessory contigs. **(g)** Genome overview of Cnym31, red line in Chr1 indicates *Starship* Ph1H2. **(h)** Genome feature PCA on gene-, TE-, GC-content and coverage and **(i)** accessory contigs. **(j)** Genome overview of Cnym26, red line in Chr1 and 9 indicates *Starship* Ph1H1 and Pr1H8, respectively. **(k)** Genome feature PCA on gene-, TE-, GC-content and coverage and **(l)** accessory contigs. **(m)** Genome overview of Cnym28. **(n)** Genome feature PCA on gene-, TE-, GC-content and coverage and **(o)** accessory contigs.


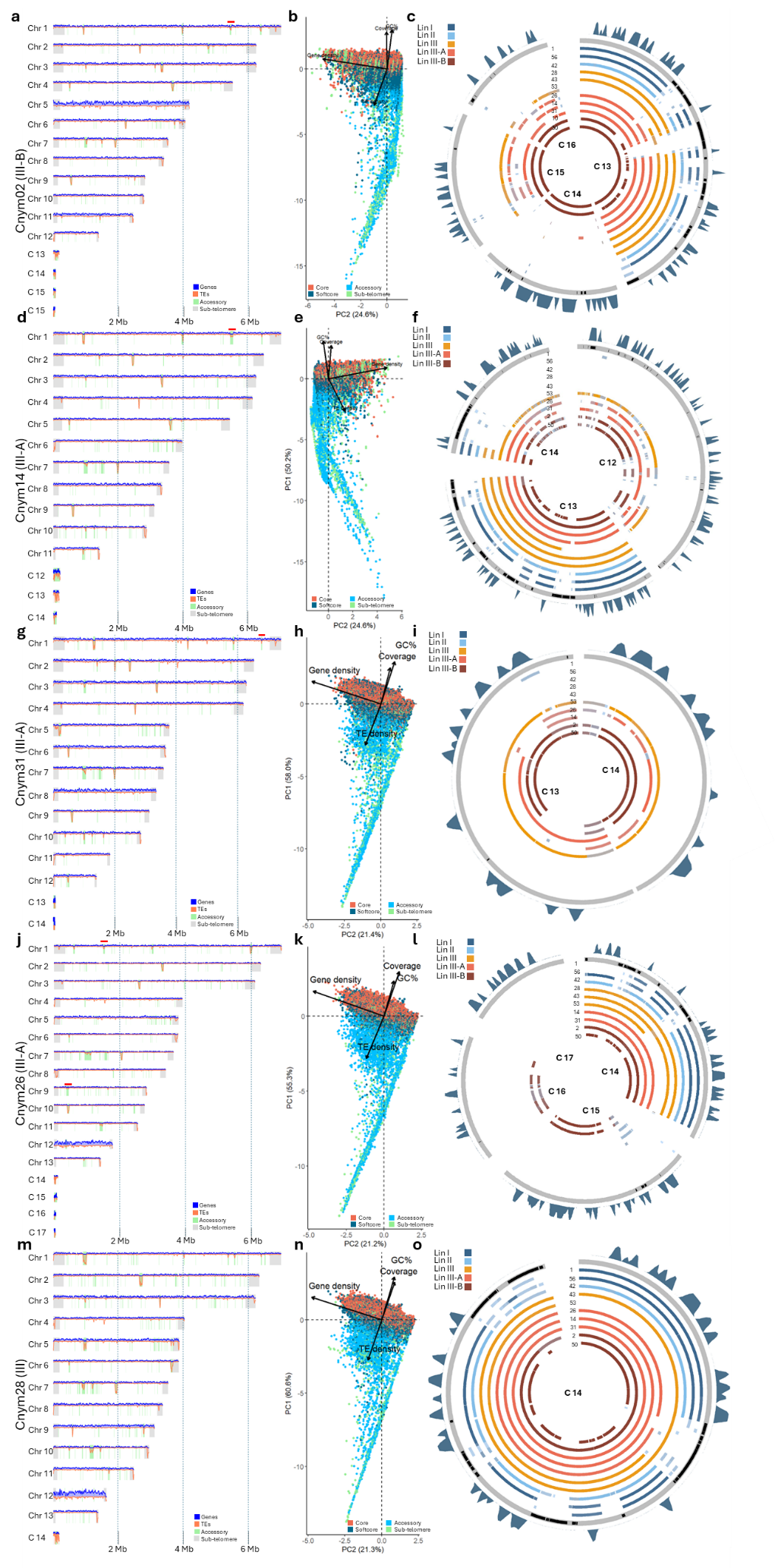


**Fig. S17** **Clustering based on accessory orthologous group (OG) gene count of *Colletotrichum nymphaeae*.** Clustering conforms the described *C. nymphaeae* clonal lineages.

**
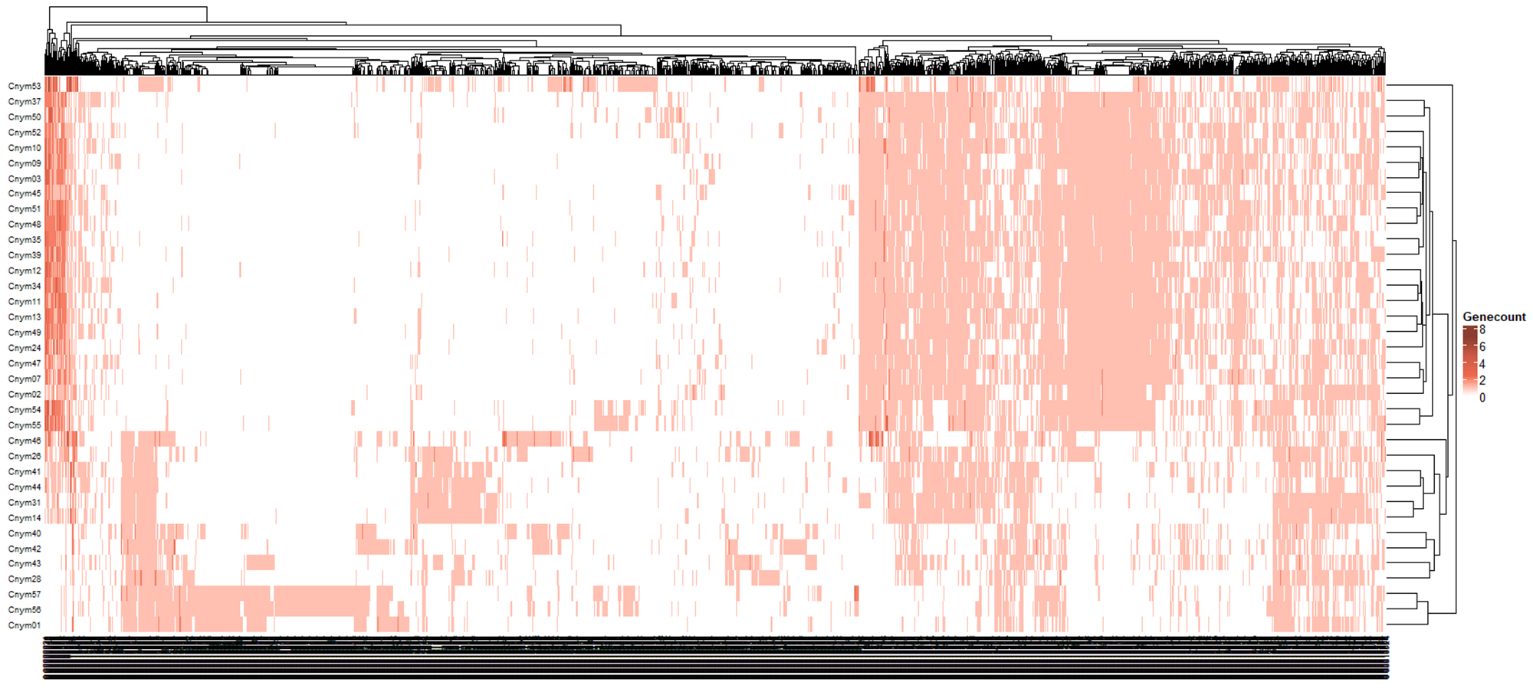
**

**Fig. S18** **De-repression of transposable element (TE) superfamilies during strawberry leaf and fruit infection. (a)** TE de-repression on leaves and **(b)** fruit 5 days post inoculation (dpi) with reads called on Cnym01 (I). **(c)** TE de-repression on leaves and **(d)** fruit 5 dpi with reads called on Cnym02 (III-B). Gray dot within boxplot indicates the mean.

**
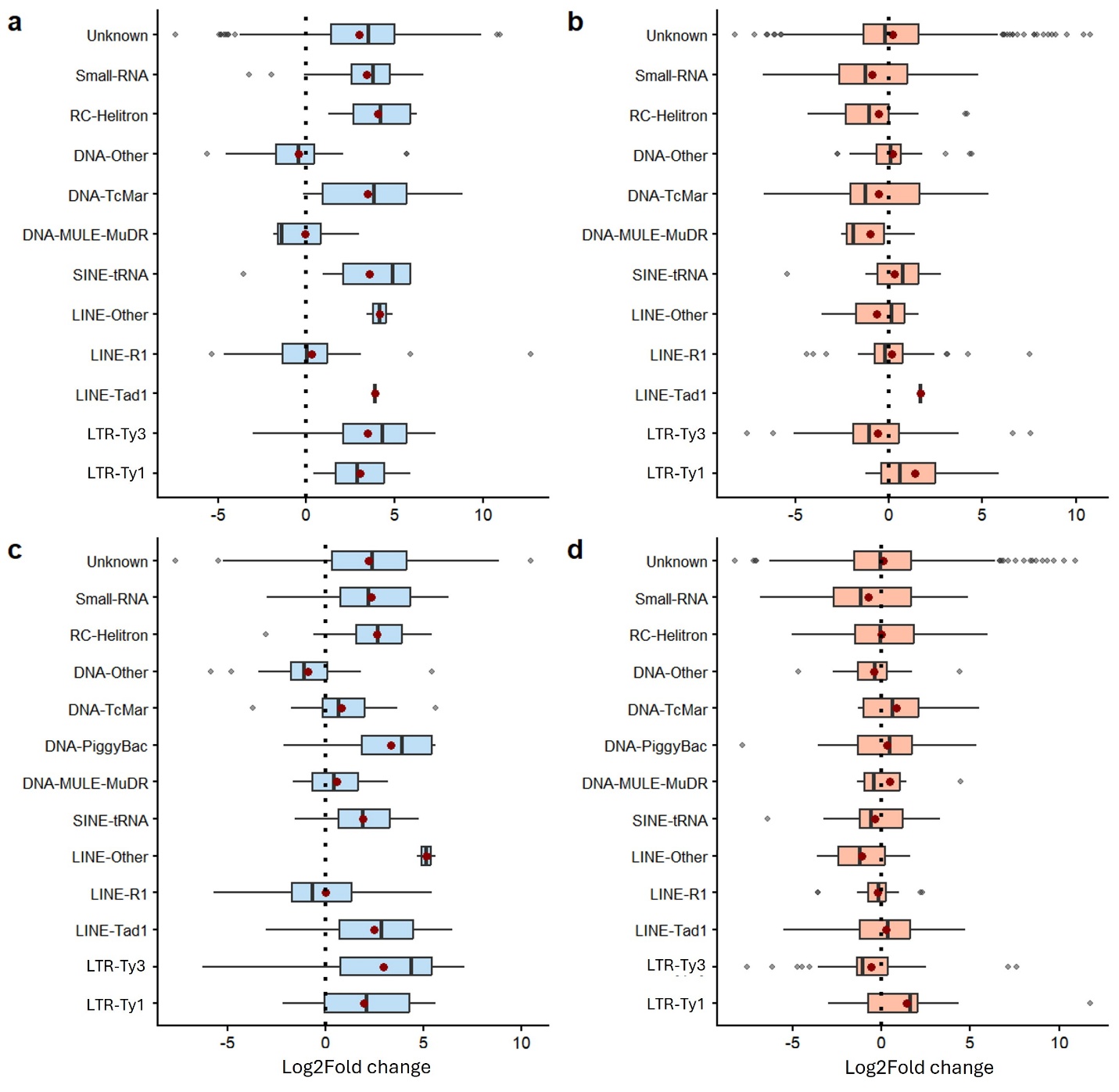
**

**Fig. S19** **Expression of gene categories during strawberry leaf and fruit infection. (a)** Expression of all genes per gene category on strawberry fruit (red, left) and leaves (blue, right) 5 days post inoculation, **(b)** expression of genes with different expression levels (p < 0.05) with reads called on Cnym01 (I). **(c)** Expression of all genes per gene category on strawberry fruit (red) and leaves (blue) 5 days post inoculation, **(d)** expression of genes with different expression levels (p < 0.05) with reads called on Cnym02 (III-B). Black dot within violin plot indicates the mean.

**
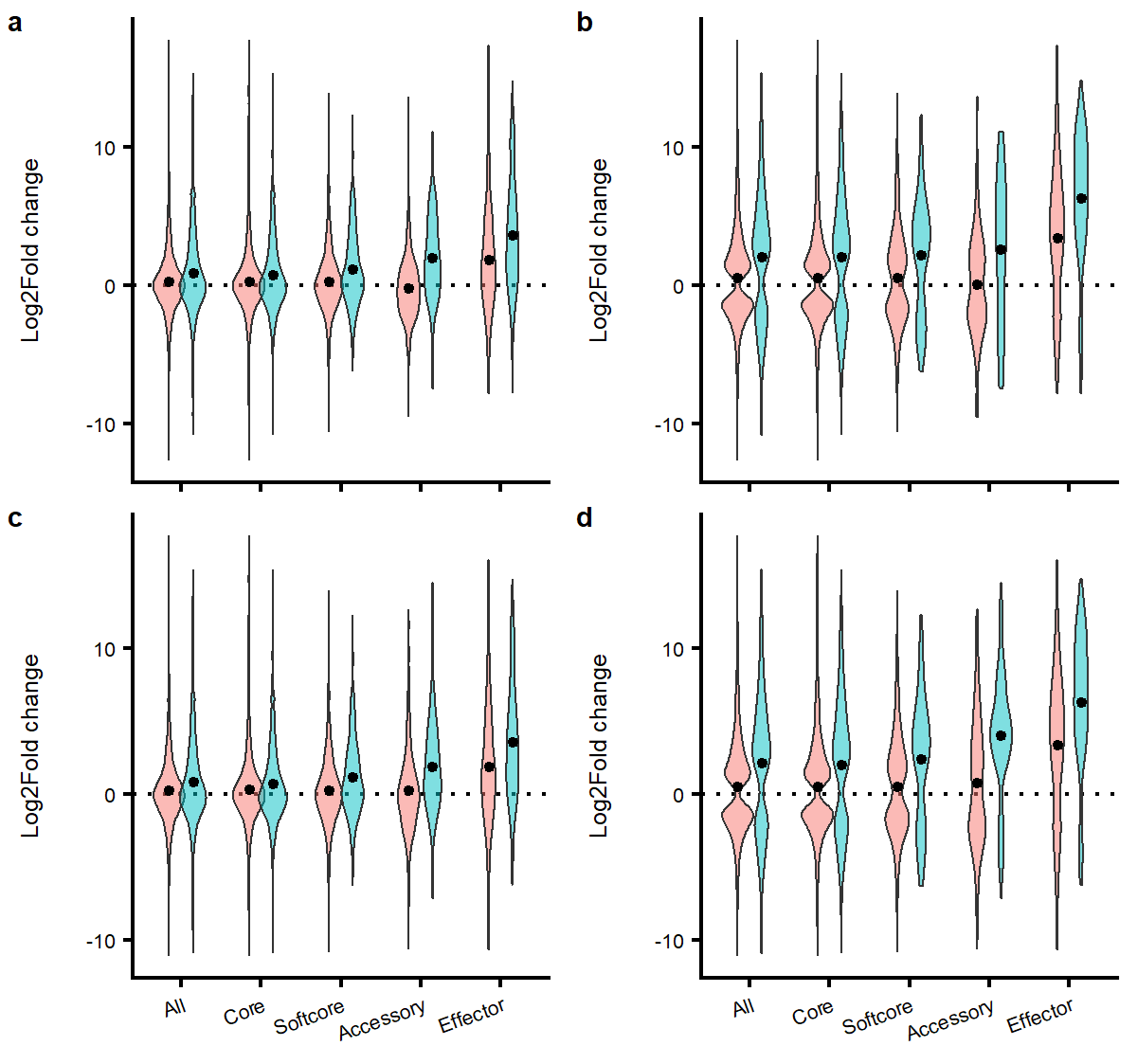
**

**Fig. S20** **Expression per chromosome during strawberry leaf and fruit infection. (a)** Expression of all genes per chromosome on strawberry fruit (red, left) and leaves (blue, right) 5 days post inoculation, **(b)** expression of genes with different expression levels (p < 0.05) with reads called on Cnym01 (I). **(c)** Expression of all genes chromosome on strawberry fruit (red) and leaves (blue) 5 days post inoculation, **(d)** expression of genes with different expression levels (p < 0.05) with reads called on Cnym02 (III-B). Black dot within violin plot indicates the mean.

**
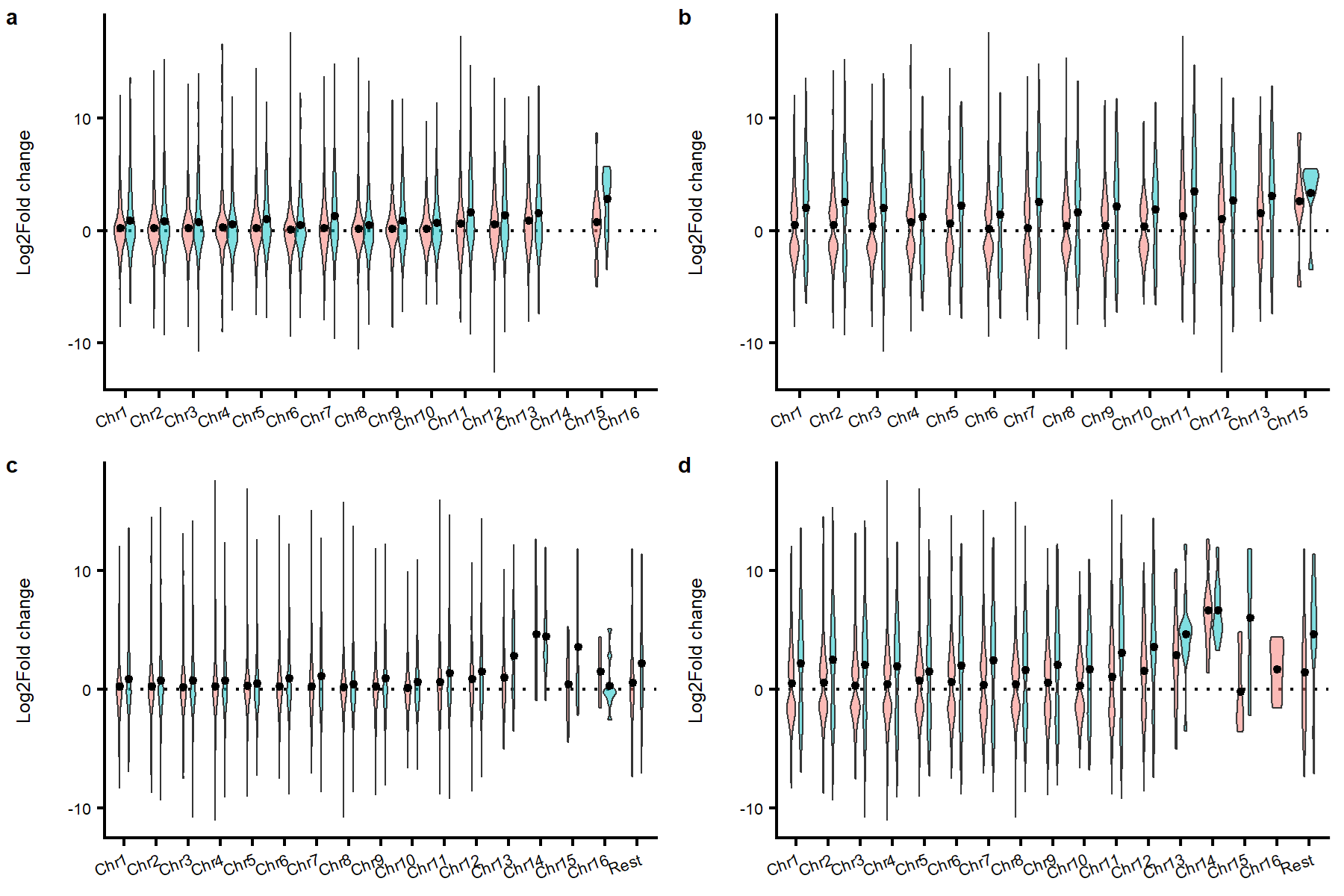
**


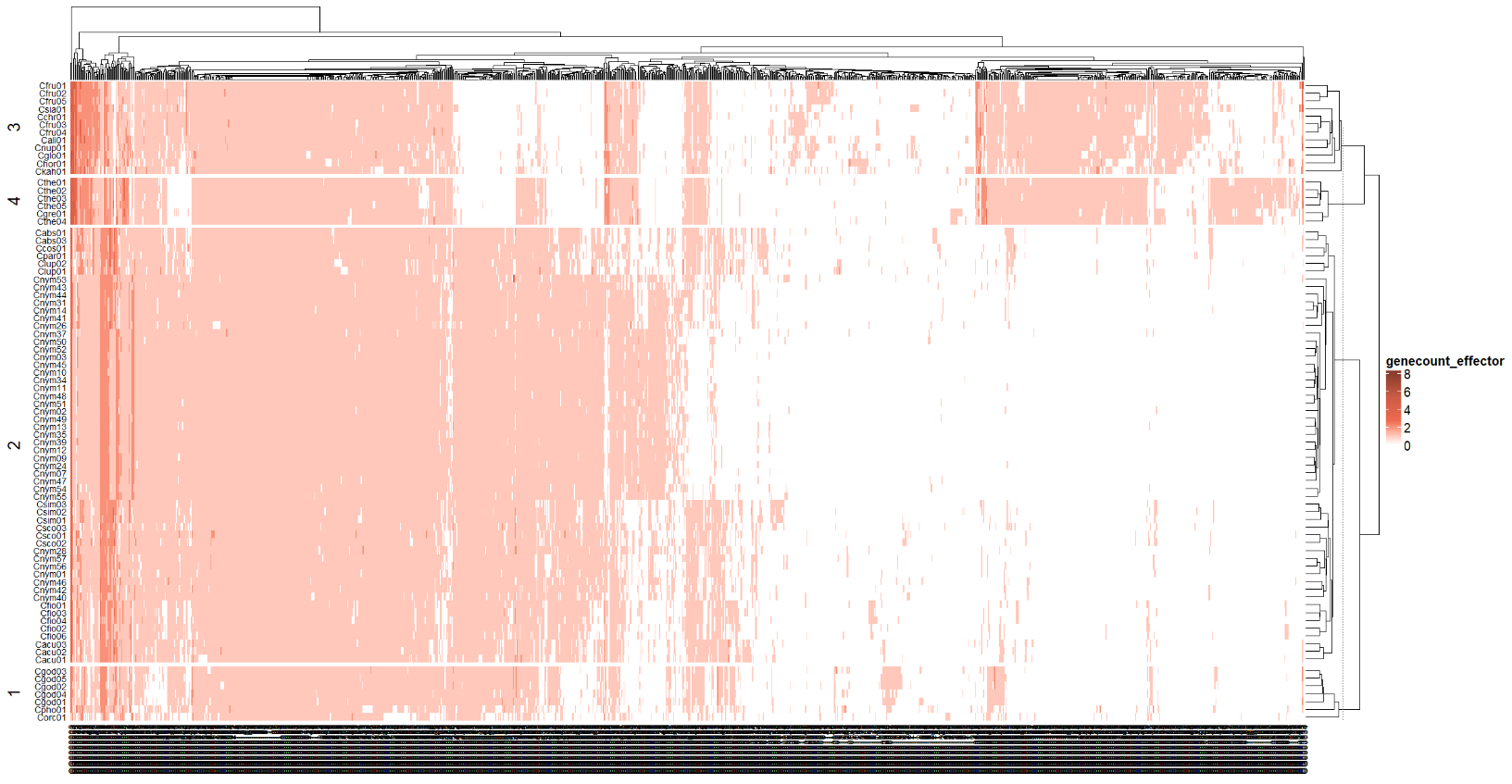
**Fig. S21** **Clustering based on effector orthologous group (OG) gene count of *Colletotrichum*.**

**Fig. S22** **Gene trees of (a) OG0603 and (b) OG0604.** Red indicates *Colletotrichum nymphaeae* lineage III-B and green represents members of the *C. gloeosporioides* species complex (*Cg*SC). See **Table S5** for predicted gene function.

**
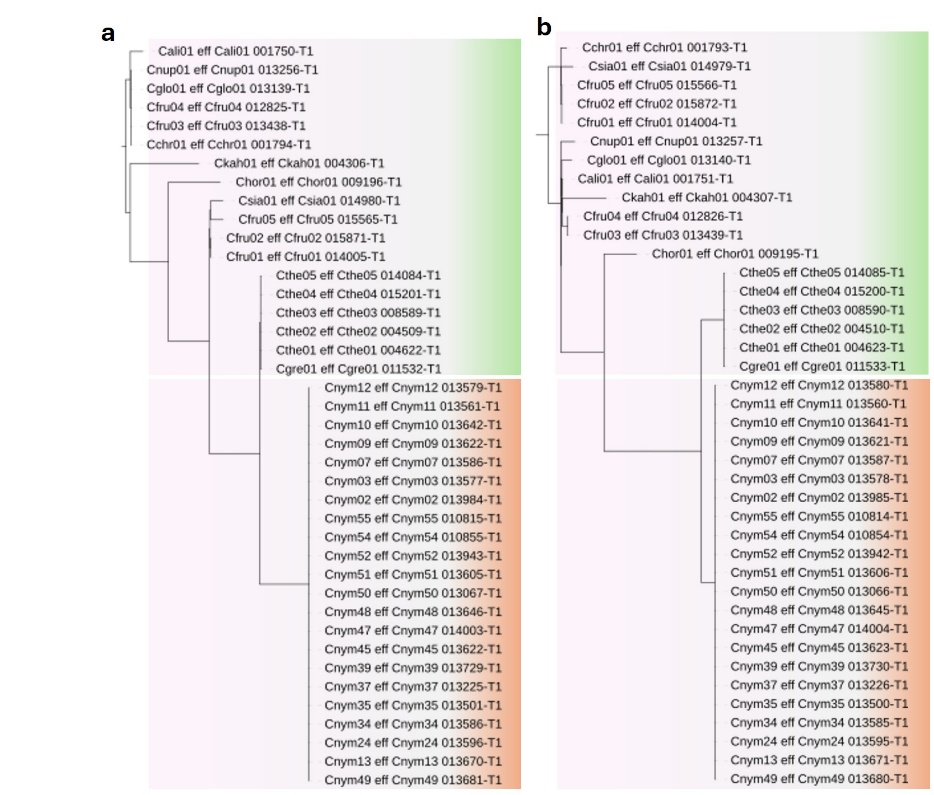
**

**Table S1 Isolate and genome details.**

**Table S2 Transcriptomic data used in this study.**

**Table S3 Lineage and population diversity statistics.**

**Table S4 *Colletotrichum Starships* identified in this study.**

**Table S5 Functional information of predicted species/lineage specific effectors and accessory region related genes.**

**Table S6 Variants associated with temporal change and their associated candidate genes, TEs and protein sequences.**
